# Comparison of two methods for the estimation of COVID-19 vaccine effectiveness of the autumnal booster within the VEBIS-EHR network in 2022/23

**DOI:** 10.1017/S0950268825000317

**Published:** 2025-03-17

**Authors:** Susana Monge, James Humphreys, Nathalie Nicolay, Toon Braeye, Izaak Van Evercooren, Christian Holm Hansen, Hanne-Dorthe Emborg, Massimo Fabiani, Chiara Sacco, Jesús Castilla, Iván Martínez-Baz, Brechje de Gier, Susan Hahné, Hinta Meijerink, Anja Bråthen Kristoffersen, Ausenda Machado, Patricia Soares, Mario Fontán-Vela, Anthony Nardone, Esther Kissling, Baltazar Nunes, VEBIS-Lot 4 working group

**Affiliations:** 1 Department of Communicable Diseases, National Centre of Epidemiology, Institute of Health Carlos III, Madrid, Spain; 2 CIBER on Infectious Diseases, Madrid, Spain; 3 Department of Epidemiology, Epiconcept, Paris, France; 4 Vaccine Preventable Diseases and Immunisation, European Centre for Disease Prevention and Control (ECDC), Solna, Sweden; 5 Department of Epidemiology and Public Health, Sciensano, Elsene, Belgium; 6 Department of Infectious Disease Epidemiology and Prevention, Statens Serum Institut, Copenhagen, Denmark; 7 Infectious Diseases Department, Istituto Superiore di Sanità, Rome, Italy; 8 European Programme on Intervention Epidemiology Training (EPIET), European Centre for Disease Prevention and Control, Stockholm, Sweden; 9 Instituto de Salud Pública de Navarra – IdiSNA, Pamplona, Spain; 10 CIBER on Epidemiology and Public Health, Madrid, Spain; 11 Center for Infectious Disease Control, National Institute for Public Health and the Environment (RIVM), Bilthoven, The Netherlands; 12 Department of Infection Control and Vaccines, Norwegian Institute of Public Health (NIPH), Oslo, Norway; 13 Department of Method Development and Analytics, Norwegian Institute of Public Health (NIPH), Oslo, Norway; 14 Departamento de Epidemiologia, Instituto Nacional de Saúde Doutor Ricardo Jorge, Lisboa Portugal; 15 Public Health and Epidemiology Research Group, School of Medicine and Health Sciences, Universidad de Alcalá, Alcalá de Henares, Spain

**Keywords:** cohort design, COVID-19, electronic health records, hospitalization, multi-country study, SARS-CoV-2, vaccine effectiveness

## Abstract

Within an infrastructure to monitor vaccine effectiveness (VE) against hospitalization due to COVID-19 and COVID-19 related deaths from November 2022 to July 2023 in seven countries in real-world conditions (VEBIS network), we compared two approaches: (a) estimating VE of the first, second or third COVID-19 booster doses administered during the autumn of 2022, and (b) estimating VE of the autumn vaccination dose regardless of the number of prior doses (autumnal booster approach). Retrospective cohorts were constructed using Electronic Health Records at each participating site. Cox regressions with time-changing vaccination status were fit and site-specific estimates were combined using random-effects meta-analysis. VE estimates with both approaches were mostly similar, particularly shortly after the start of the vaccination campaign, and showed a similar timing of VE waning. However, autumnal booster estimates were more precise and showed a clearer trend, particularly compared to third booster estimates, as calendar time increased after the vaccination campaign and during periods of lower SARS-CoV-2 activity. Moreover, the decrease in protection by increasing calendar time was more clear and precise than when comparing protection by number of doses. Therefore, estimating VE under an autumnal booster framework emerges as a preferred method for future monitoring of COVID-19 vaccination campaigns.

## Key results

- Vaccine Effectiveness (VE) was mostly similar using two methodological approaches: accounting vs. not accounting for the total number of booster doses, with open or closed cohorts, respectively, highlighting the low practical implications of distinguishing VE by the number of boosters.
- VE of the autumnal booster dose regardless of the number of prior boosters (autumnal approach) provided more precise results with a clearer gradually decreasing trend with increasing calendar time, compared to third booster-specific VE.
- The robustness and precision of the autumnal booster approach were more evident as time elapsed after the vaccination campaign and during periods of lower SARS-CoV-2 activity.
- Estimating VE of the 2023 autumnal booster under an autumnal booster framework (without accounting for previous doses) emerges as a preferred method for future monitoring of COVID-19 vaccination campaigns.

## Introduction

Since the start of the coronavirus disease 2019 (COVID-19) vaccination campaigns in December 2020, monitoring the vaccine effectiveness (VE) has been key to guide decision-making on vaccination policies [1, 2], which have been constantly adapted to confer the highest protection against severe COVID-19 outcomes in vulnerable groups. Because of the waning protection [3–5] and emergence of SARS-CoV-2 variants with different degrees of immune escape [6–13], many countries recommended a first COVID-19 booster dose to individuals aged 65 years or older in the autumn and winter of 2021, with high effectiveness [14, 15].

In the spring of 2022, however, increased incidence of COVID-19, linked mainly to the emergence of BA.1, BA.4, and BA.5 Omicron sub-variants, led to the recommendation of a second booster in some European Union/European Economic Area (EU/EEA) countries [6, 7]. In September 2022, conditional authorization from the European Medicine Agency (EMA) was granted for bivalent vaccines targeting the original strain of SARS-CoV-2 and Omicron subvariant BA.1 or targeting the original strain of SARS-CoV-2 and the BA.4/5 omicron subvariants. These were deployed as second or third boosters in EU/EEA countries during the autumn of 2022.

The effectiveness of second and third boosters has been reported to be lower and decline faster during the BA.4 and BA.5 dominating period, even with the use of adapted vaccines [2, 6–8, 16–22], as well as during the subsequent period with dominant circulation of BQ.1 and XBB.1.5 subvariants [5, 9, 11, 21, 23, 24].

Recent VE studies have indicated that, in the current scenario, the time since the last vaccination dose is more relevant for protection than the total number of vaccine boosters [5, 25]. Therefore, methodological approaches similar to those for the monitoring of seasonal influenza vaccine effectiveness [26–28], considering exposure of interest the vaccination in the current season (even though some confounding may exist due to vaccination in the previous seasons), has been suggested as a possible way forward for COVID-19 VE monitoring, in situations in which COVID-19 vaccination is implemented as a seasonal campaign.

Since 2021, the European Centre for Disease Prevention and Control (ECDC) has funded the Vaccine Effectiveness, Burden and Impact Studies of COVID-19 and Influenza (VEBIS) project to estimate VE in real-world conditions in a multi-country approach using electronic health records (VEBIS-EHR) [29–31]. Including data on disease events from several EU/EEA countries enhances the representativeness and statistical power of VE estimations. Moreover, multi-country studies allow for the comparison of VE for the same booster dose rolled out in different target groups and at different times [16].

Our objective was to estimate the VE of booster doses against hospitalization due to COVID-19 and COVID-19 related death in seven EU/EEA countries between November 2022 and July 2023 and, specifically, to compare estimates using two methodological approaches for VE estimation. The first approach was to estimate VE for the first, second, or third COVID-19 booster dose (following similar methods to those previously used by this network), using an open cohort. The second approach estimated the VE of the vaccine dose given as part of the 2022 autumn COVID-19 vaccination campaign (“autumnal booster”), regardless of the number of prior doses received, using a closed cohort approach. A comparison of these results will support interpreting VE estimates within the current COVID-19 context and may help inform decisions on the most suitable method for future COVID-19 VE monitoring in Europe.

## Methods

*Study design and setting:* The VEBIS multi-country study uses electronic healthcare databases for the monitoring of COVID-19 vaccine effectiveness in real time.

Seven countries participated in the study: Belgium, Denmark, Italy, Norway, Portugal, the Netherlands, and Spain (Navarre). The study period was from November 2022 to July 2023. Roll-out of the second booster doses (Annex I, Supplementary material) started in spring 2022 in the Netherlands (February–March, for ≥60-year-olds), Italy (April, for ≥80-year-olds, and July, for 65–79-year-olds), Portugal (May, for ≥80-year-olds), Norway (June, for ≥65-year-olds) and Belgium (July, for ≥80-year-olds); and in autumn 2022, in Denmark (September–October, for ≥65-year-olds), and in Spain (October, for ≥65-year-olds). In September 2022, Portugal and Belgium extended the recommendation for a second booster to 65–79-year-olds and recomended a third booster dose of bivalent mRNA vaccines in ≥80 year-olds who had accepted a previous second booster in spring 2022. An additional autumnal dose was also recommended in the Netherlands (September–October for ≥60 year-olds) and Italy (in October, for ≥60 year-olds).

Retrospective cohorts were constructed at each study site using the participating site EHR. All registries had national coverage except in Belgium, where only a subset of all hospitals contributed to the registry, and in Spain, where the whole region of Navarre was included. Individual deterministic linkage was used to cross-match administrative databases with registries for COVID-19 vaccination, SARS-CoV-2 testing, hospitalizations, and, in some instances, cases reported to epidemiological surveillance systems. VE was estimated at each site by applying common protocols [29,31], and estimates were then pooled for an overall VE.

We prepared monthly VE estimates to provide near real-time monitoring. To accumulate sufficient events to support VE estimation, each monthly estimate covered an observation period of 8 weeks, with a lag of one month between the month of analysis and the end of the observational period, to allow for data consolidation (i.e. estimates produced in February 2023 covered November–December 2022). The observation period was moved one month forward for each successive monthly estimate.

### Eligibility criteria, definitions, and follow-up for the two methodological approaches

The study included individuals aged 65 years or older who had completed primary vaccination (administered no less than 19 days apart for vaccines requiring two doses for primary vaccination) with a vaccine approved by the EMA. In Navarre, unvaccinated or partially vaccinated individuals were also included.

Outcomes of interest were: (a) hospitalization due to COVID-19, defined as a hospital admission due to a severe acute respiratory infection with a SARS-CoV-2 positive test from 14 days before to 1 day after admission or as COVID-19 as the main diagnosis in admission or discharge records, except in the Netherlands, where admissions with a positive SARS-CoV-2 test and missing or unknown reason for admission (about 50% of all admissions with a positive SARS-CoV-2 test) were also included, and (b) COVID-19-related death, defined: in Norway, as death for which COVID-19 is recorded as the cause or underlying cause of death (even with no positive SARS-CoV-2 test recorded); in Navarre, as death due to laboratory-confirmed SARS-CoV-2 infection according to the medical doctor revision of clinical records; in Denmark and Italy, as laboratory-confirmed SARS-CoV-2 infection with death in the 30 days after the positive test or symptom onset and; finally, in Portugal as both deaths with COVID-19 as the cause of death and deaths with laboratory-confirmed infection in the previous 30 days. Belgium and the Netherlands did not contribute to COVID-19-related death outcomes.

Two different methodological approaches were implemented, with vaccination status included as time-varying exposure. The first approach [29] defined an open cohort where eligibility was verified at the start of each different observation period. Eligible individuals were those who were ≥ 65 years of age and had completed primary vaccination a minimum of 168 days ago. Individuals were dynamically classified (i.e. allowing them to change vaccination status during the study period follow-up) into no booster, first, second, or third booster dose group. The status was considered achieved 14 days after administration of the corresponding booster, separated a minimum of 90 days from any previous dose. First, second, and third booster VE was estimated, using as reference for comparison those with primary vaccination but no booster. This has been the approach used by the VEBIS-EHR network up to July 2023 [5, 16, 25, 32].

The second approach [31] defined a closed cohort where eligibility was verified at the start of the study site-specific 2022 autumnal vaccination campaign, with no later entry of individuals into the study. Eligible individuals were those who were ≥ 65 years of age and who had completed primary vaccination a minimum of 180 days and had a minimum of 90 days since any previous COVID-19 vaccine dose or any previously documented SARS-CoV-2 infection (even if the recommended interval between doses was higher in some participating sites, such as Italy, where the booster was recommended to those with no vaccine dose in the previous 180 days). Individuals were dynamically classified as vaccinated with the autumnal booster after 14 days of receipt of the booster dose. Individuals were censored when receiving any additional dose after the autumnal booster. Autumnal booster recipients were compared to individuals eligible for a booster but who had not yet received it, to estimate autumnal booster VE.

In both approaches, the time since the booster dose (first, second, or third booster dose in the first approach or autumnal booster in the second) was split into days from 14 to 89, 90 to 179 and 180 or more days since booster receipt. Individuals may dynamically transition between statuses during follow-up, and VE for each status was calculated relative to the same reference group used in the overall models.

Individuals were followed up from the first day of each observation period up to the earliest occurrence of any of the following events: (a) the outcome of interest, (b) discontinuation in the administrative database (e.g. emigration), (c) death of any cause, and (d) the end of the observation period. In the second approach, only the first outcome in the season was counted and individuals were also censored if they received an additional dose after the autumnal booster or upon receiving any vaccine dose after the end of the vaccination campaign.

### Statistical analysis

We used Cox proportional hazards regression models, assigning time zero to the first day of each observation period. We estimated adjusted hazard ratios (aHR) and 95% confidence intervals (CI) by sex, age group (in 5-year age bands), territorial division (as appropriate in each study site), previous SARS-CoV-2 infection (only in the first approach), comorbidities, number of previous vaccine doses (only in the second approach) and other variables as relevant at each site (see Annex 2, supplementary material, for further details on adjustment variables and variables definitions at each site). Only sites with general recommendations for the respective doses in 65–79 years and ≥ 80 years old were included in each 8-week observation period for each age group. Site-specific aHR estimates were pooled using Paule-Mandel random-effect meta-analysis [33], and VE was derived as VE = (1 – aHR) × 100.

For data protection reasons, sites reported aHR estimates only when at least five events (ten events in the Netherlands) per vaccination status category were observed. Pooled VE estimates were not reported where they were based on fewer than 15 events across all pooled sites. All sites fulfilled ethical and data protection requirements according to their national legislation (Annex 3, supplementary material).

## Results

### Study participants

Among the seven study sites, in each 8-week study period, between 18.5 and 25.4 million people ≥65 years old were recruited, adding up to between 33.7 and 44.9 million person-months of follow-up (Annex 4, supplementary material). In the first approach, the proportion of person-time with a second booster increased from 40% to 43% from the first to the last study period, with a third booster, from 5% to 6%. In the second approach, the proportion with the autumnal booster was 41% in the first study period and 53% in the last.

We evaluated the characteristics of the sample by pooling descriptive data from all study periods and sites. In the first approach, the proportion of person-time contributed by ≥80-year-olds with medium or high-risk comorbidities (see Annex 2 for the full list of included conditions) was higher among those with a first, second, or third booster (32%, 43%, and 55%, respectively, with medium-risk comorbidities vs. 28% from individuals with no booster; and 2%, 4%, and 9%, respectively, with high-risk comorbidities vs. 2% from individuals with no booster). Among 65–79-year-olds, proportions with medium-risk comorbidities were 33% and 48% for people with one or two boosters vs. 28% in people with no booster; and 2% and 6%, respectively, with high-risk comorbidities versus 2% in those with no booster. In the second approach, 59% and 7% of ≥80-year-olds and 47% and 6% of 65–79-year-olds who received the seasonal booster had medium and high comorbidities, respectively, compared to 35% and 3% in ≥80-year-olds and 35% and 2% in 65–79-year-olds who did not receive it. Sample characteristics by study site, sex, nationality, country of birth, number of booster doses, and type of vaccine received are available in Annex 5 of the Supplementary material.

Bivalent vaccines comprised 60% and 97% of second and third boosters respectively, but 96% of 2023 autumnal vaccines. Bivalent vaccines represented 43% and 67%, in ≥80-year-olds and 65–79-year-olds, respectively, of all second boosters by the end of the study follow-up. Among the autumnal vaccines, BA.4/5 and BA.1 were equally distributed (48% each). Of these, 83% were from Pfizer and 17% from Moderna.

We present below the estimates of VE against hospitalization due to COVID-19, while mostly similar conclusions are reached with estimates of VE against COVID-19-related death (Annex 6 and Annex 7, Supplementary material).

### First methodological approach: VE by number of booster doses

Between November 2022 and July 2023, a first booster provided little to no added protection compared to complete primary vaccination only (≥168 days ago). Between November–December 2022 and June–July 2023, estimates ranged between 23% (95% CI: −4; 42) and 15% (95% CI: −38; 48) and between 34% (95% CI: 25; 42) and 27% (95% CI: 4; 44) in ≥80-year-olds and 65–79 year-olds, respectively (Tables 1 and 2).

**Table 1.** Vaccine effectiveness (95% confidence intervals) in those aged ≥80 years against hospitalization due to COVID-19 according to two approaches. Protocol v1.0: vaccine effectiveness (VE) of the first, second, and third booster dose, compared to complete primary vaccination without booster administered ≥168 days ago. Protocol v2.0: autumnal (bivalent) vaccine effectiveness among individuals eligible for an annual vaccine. For each 8-week overlapping study period between November 2022 and July 2023

Study period	Protocol version 1.0			Protocol v2.0
	Complete primary vaccination + first booster dose	Complete primary vaccination + two booster doses	Complete primary vaccination + three booster doses^a^	Autumn booster vaccination
1st November to 26th December 2022	22.5% (−3.7; 42.1)	60.0% (31.9; 76.5)	56.7%^a^ (41.8; 67.9)	61.4% (54.9; 67)
1st December 2022 to 25th January 2023	19.1% (−10.4; 40.7)	51.2% (23.6; 68.8)	47.3%^a^ (25.7; 62.6)	53.3% (46.3; 59.5)
1st January to 25th February 2023	15.6% (−22.3; 41.8)	40.1% (12.5; 58.9)	30.0%^a^ (2.4; 49.8)	40% (33.8; 45.6)
1st February to 28th March 2023	−4.8%^c^ (−35.1; 18.7)	21.6% (−11.0; 44.6)	7.5%^a^ (−70.8; 49.9)	28% (21.9; 33.7)
1st March to 25th April 2023	11.9%^c^ (−25.1; 38.0)	25.0% (−15.5; 51.3)	5.0% (−93.4; 53.4)	25.7% (20.1; 30.9)
1st April to 26th May 2023	13.1%^c^ (−28.6; 41.3)	27.8% (−12.2; 53.5)	24.2% (−45.8; 60.6)	22.2% (13.3; 30.1)
1st May to 25th June 2023	18.3%^c^ ^,^^d^ (−52.3; 56.1)	16.6%^c^ (−34.8; 48.4)	36.6%^b^ (−67.9; 76)	19.9% (1.5; 34.9)
1st June to 26th July 2023	14.9%^b^ ^,^^c^ ^,^^d^ (−38.1; 47.6)	17.4%^b^ (−23.9; 44.9)	33.9%^b^ (−39.0; 68.6)	16.2% (1.1; 28.9)

a Denmark and Navarra (Spain) did not recommend a third booster for individuals aged ≥80 years during the study period and, therefore, do not contribute to third booster estimates. Norway recommended it in March 2023 and thus contributed only from March–April onwards.

b Belgium did not reach 5 events and estimates were not provided.

c Denmark did not reach 5 events and estimates were not provided.

d Navarra (Spain) did not reach 5 events and estimates were not provided

**Table 2.** Vaccine effectiveness (95% confidence intervals) in those aged 65 to 79 years against hospitalization due to COVID-19 according to two approaches. Protocol v1.0: vaccine effectiveness (VE) of the first, second, and third booster dose, compared to complete primary vaccination without booster administered ≥168 days ago. Protocol v2.0: autumnal (bivalent) vaccine effectiveness among individuals eligible for an annual vaccine. For each 8-week overlapping study period between November 2022 and July 2023

Study period	Protocol version 1.0			Protocol v2.0
	Complete primary vaccination + first booster dose	Complete primary vaccination + two booster doses	Complete primary vaccination + three booster doses^a^	Autumn booster vaccination
1st November to 26th December 2022	34.1% (24.9; 42.2)	68.3% (52.6; 78.8)	54.2% (23.2; 72.7)	64.7% (57.3; 70.9)
1st December 2022 to 25th January 2023	35.1% (21.8; 46.2)	64.2% (42.7; 77.6)	57.0% (39.3; 69.5)	55.7% (45.5; 64.1)
1st January to 25th February 2023	33.7% (26.5; 40.3)	56.6% (44.5; 66.1)	55.5% (37.1; 68.5)	42.5% (37.6; 47.1)
1st February to 28th March 2023	22.1% (12.5; 30.7)	39.1% (31.3; 46.1)	43.7% (21.5; 59.6)	43% (33.7; 51)
1st March to 25th April 2023	−271.5% (-Inf; 82.8)	43.1% (28; 55.1)	37.5% (0.8; 60.7)	34.8% (29.6; 39.6)
1st April to 26th May 2023	13.7% (0.7; 25.0)	39.8% (17.0; 56.3)	41.5% (0.6; 65.6)	29.8% (23.2; 35.9)
1st May to 25th June 2023	11.3%^b^ ^,^^c^ ^,^^d^ (−7.6; 27.0)	21.5%^c^ ^,^^d^ (3.7; 36.0)	19.9% (−36.2; 52.8)	29.8% (18.4; 39.7)
1st June to 26th July 2023	27.1%^b^ ^,^^c^ ^,^^d^ (4.2; 44.4)	26.9%^b^ ^,^^c^ (3.0; 44.9)	4.1% (−58.7; 42.1)	22.4% (6.8; 35.4)

a A third booster dose in the group 65 to 79 years was only recommended in Italy and the Netherlands, therefore all estimates are based on only these two study sites.

b Belgium did not reach 5 events and estimates were not provided.

c Denmark did not reach 5 events and estimates were not provided.

d Navarra (Spain) did not reach 5 events and estimates were not provided.

**Figure 1.**
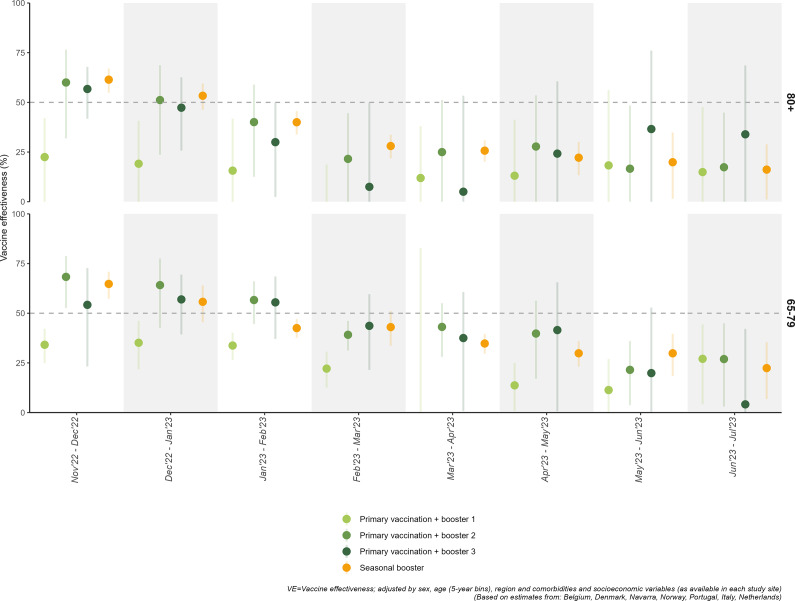
Vaccine effectiveness (95% confidence intervals) against hospitalization due to COVID-19 according to two approaches. Protocol v1.0: vaccine effectiveness (VE) of the first, second, and third booster dose, compared to complete primary vaccination without booster administered ≥168 days ago. Protocol v2.0: autumnal (bivalent) vaccine effectiveness among individuals eligible for the autumnal vaccine dose. For each 8-week overlapping study period between November 2022 and July 2023.

The VE of a second booster was high shortly after administration (November–December 2022): 60% (95% CI: 32; 77) overall and 66% (95% CI: 45; 78) within 90 days after vaccination in ≥80-year-olds and 68% (95% CI: 53; 79) overall and 73% (95% CI: 65; 79) within 90 days after vaccination in 65–79-year-olds. The VE declined during the study period (Figure 1) and by time since vaccination (Annex 6, supplementary material), with VE estimates <50% from January–February 2023 onwards for the ≥80-year-olds and from February–March onwards for the 65–79-year-olds. Low residual protection was observed in June–July 2023 in the ≥80-year-olds (17%; 95% CI: −24; 45) and in the 65–79-year-olds (27%; 95%CI: 3; 45).

The VE of a third booster administered during autumn 2022 could only be estimated in those groups who received the second booster vaccination during the spring 2022 campaign in Belgium, Italy, Portugal, and the Netherlands. In November–December 2022, the VE of the third booster was 57% (95% CI: 42; 68) in ≥80-year-olds and 54% (95% CI: 23; 73) in 65–79-year-olds, similar to the VE of a second booster in the same period (Tables 1 and 2). The VE of third boosters waned rapidly, falling below 50% from December–January onwards in ≥80-year-olds and from February March onwards in 65–79-year-olds. By the time since vaccination (Annex 6, Supplementary material), low VE was estimated beyond 90 days of administration. In the last period available (June–July 2023), the VE of the third booster was 34% (95% CI: −39; 69) in ≥80-year-olds and 4% (95% CI: −59; 42) in 65–79-year-olds.

### Second methodological approach: VE of the seasonal booster

From November 2022 to June 2023, autumnal booster VE among the ≥80-year-olds decreased steadily from 61% (95%CI: 55; 67) to 16% (95%CI: 2; 35), achieving protection lower than 50% from January–February 2023 onwards (Table 1). A similar pattern was observed in the 65–79-year-olds, in whom VE was 65% (95%CI: 57; 71) at the start of the campaign (November–December 2022), and went below 50% from January–February 2023 onwards, down to 22% (95%CI: 7; 35) in the last study period (June–July 2023) (Table 2).

In all reporting periods, we observed a decrease in VE with time since vaccination (Annex 6, Supplementary material). During the first periods (November 2022–January 2023), this decrease was of small magnitude. However, in the following months VE decreased even within the first 90 days or on days 90–179 after vaccination, decreasing from November–December 2022 (respectively, 63% and 62% in the ≥80-year-olds and 66% and 58% in the 65–79-year-olds) to March–April 2023 (respectively, −2% and 26% in the ≥80-year-olds and 51% and 37% in the 65–79-year-olds). VE achieved low levels of protection for those with 180 or more days since vaccination, of 7% in the ≥80-year-olds and 23% in the 65–79-year-olds in the last available period (June–July 2023).

## Discussion

The two methodological cohort approaches provided comparable estimates, consistent with moderate to high VE estimates, particularly at the beginning of the study period. In November and December 2022, VE estimates for the autumnal vaccine – without accounting for the previous number of doses- were similar than VE estimates for the second or third booster. This was expected as the majority of doses administered as autumnal boosters were either second (73%) or third boosters (25%). However, estimates for autumnal vaccination used as reference group individuals who may have received one or two boosters ≥90 days before the start of the campaign, potentially resulting in a lower VE estimated for the autumnal vaccination compared to using only people with complete primary vaccination but no boosters, as in the first approach. Also, a decline in protection during the study period, as well as with increasing time since vaccination, followed a similar timing in both methodological approaches, with VE dropping below 50% approximately two to three months after the study began. However, the decline was slower, and the precision of the estimates was higher using the second method.

Particularly, the greater decline of VE for the third booster dose was not observed in the autumnal vaccine approach. Third boosters were administered to individuals who had received all recommended doses in countries that had rolled out spring vaccination only about six months earlier. Individuals accepting a third dose were probably highly vulnerable and had a lower probability of previous SARS-CoV-2 infections (compared to others who may have declined some vaccine dose due to ongoing or recent SARS-CoV-2 infection at the time of recommendation). This would increase their higher background risk and make them less comparable to the reference group (those with complete primary vaccination only) in ways difficult to account for. As previous SARS-CoV-2 infections have mostly been detected by self-testing since the Omicron variant became dominant in early 2022, EHRs are considered an incomplete data source of documented infections. On the other hand, although we adjusted for comorbidities, it is improbable that we completely captured the complex conditions that lead to the likelihood of accepting vaccination. Therefore, the autumnal vaccination approach reduces the likelihood of selecting higher-risk individuals in a single category (e.g. individuals receiving a third booster) and, in so doing, may result in estimates less affected by strong confounding bias. Finally, our results for the autumnal booster were adjusted by the number of previous boosters at the start of the autumnal campaign. Models not accounting for this showed a relative change in VE of 4%–10%, depending on the model (results not shown), indicating confounding. Future studies will try to elucidate whether effect modification is also relevant.

On the other hand, the higher precision of the estimates for the autumnal vaccine in periods of low SARS-CoV-2 circulation –and therefore a lower number of events- was also expected. The complete sample size and number of events are categorized in only two groups (those who received or who did not receive the autumnal vaccine), as opposed to the four groups needed to monitor the effectiveness of each booster dose specifically. This reduced the level of data sparsity, leading to less random variability. As the number of possible vaccination statuses continues to grow, the greater efficiency of the autumnal vaccine approach holds significant methodological value.

Finally, the autumnal booster estimation used a closed cohort approach which ensures that all individuals included as vaccinated receive the autumnal campaign dose, as opposed to the open cohort, which could include individuals vaccinated at any time. Also, the closed cohort approach prevents individuals who completed their primary vaccination early in the season from entering the reference group in the latter periods, although this was infrequent. These aspects have probably contributed to greater stability of the autumnal VE estimates during the latter part of the study period and also provided results that were easier to interpret. Including only individuals eligible for the autumnal vaccine also increases internal validity, given that all included individuals have a non-null probability of being exposed to the autumnal vaccine. When estimating VE by number of boosters, individuals with primary vaccination only or only one booster have a null probability of being part of the third booster group during the study period, which can be seen as a violation of the positivity condition to causal inference from observational studies [34].

Some limitations of our study relate to the heterogeneity that could arise from its multi-country approach. Even though we used a common protocol across the 7 study sites, we made a secondary use of data collected for another purpose and, thus had a limited capacity to enhance data granularity or alignment of covariate definition when required. Therefore, a certain degree of methodological heterogeneity across the study is anticipated and may have affected our results. Moreover, the number of study sites contributing to the different monthly estimates can differ if, due to a low number of events, a particular site is not included for a particular study period. The exclusion of sites with <5 events could remove sites differentially depending on vaccine effectiveness, vaccine uptake, or underlying risk. Additionally, individuals with the null probability of vaccination and outcome could be included if deceased or emigrated individuals were not updated in the databases. To minimise this, we excluded unvaccinated individuals and those with incomplete vaccination, who may also differ from other population groups in ways not measured in our study. Finally, in a multi-country approach, true heterogeneity in VE may exist due to the use of different vaccines at different times in potentially distinct populations (for example, regarding their age distribution, the proportion with past infection, exposure behaviours, etc.). Nevertheless, the added value in terms of robustness, richness of data, and representativeness of such multi-country collaborations outweighs the limitations.

Overall, our results are concordant with previous estimates of VE in the same period [7, 17, 18, 21–24]. However, there is heterogeneity in the literature. In Italy, a matched cohort study estimated VE against severe COVID-19 at 51% (95%CI: 46–55) in the first four months post-vaccination [20], while using a test-negative design, the hospital network within the VEBIS study estimated a VE of 80% (95%CI: 50 to 94) against COVID-19 hospitalization at <90 days since vaccination and 15% (95%CI: −12 to 35) thereafter [11]. Available evidence supports the hypothesis that, at least since the deployment of the 2022 autumn vaccination campaign, time since the last booster dose is the main driver of VE, and not the total number of booster doses received [5, 25]. This is particularly relevant in the context of waning protection observed in our study and elsewhere [3–5], although higher residual protection was estimated when analyzing the autumnal vaccination as a whole. These results support the recommendation of additional COVID-19 vaccine boosters for the targeted age groups, regardless of previous vaccinations.

In conclusion, estimating COVID-19 VE by comparing the risk among those who received the autumnal booster dose and those eligible but who did not receive it, while not taking into account the history of previous booster doses, was more statistically powered and showed a clearer trend, particularly as more time elapsed and during periods of lower SARS-CoV-2 activity. Importantly, this method aimed to determine the effect of an intervention on individuals eligible to receive it, allowing for the translation of results into a clearer and more easily conveyed public health message. Therefore, estimating VE under an autumnal booster framework emerges as a preferred method for future monitoring of COVID-19 vaccination campaigns.

## Supporting information

Monge et al. supplementary material Monge et al. supplementary material

## Data Availability

Authors cannot share the data used for this study, which should be requested by the data owner institutions following their respective procedures.

## Supplementary material

For supplementary material accompanying this paper visit http://doi.org/10.1017/S0950268825000317. click here to view supplementary material
