# Supplementary material for: Comparison of two methods for the estimation of COVID-19 vaccine effectiveness of the autumnal booster within the VEBIS-EHR network in 2022/23
Source: Epidemiol Infect. 2025 Mar 17;153:e54. doi: 10.1017/S0950268825000317 (PMC12001144; doi:10.1017/S0950268825000317)
Supplement: Monge et al. supplementary material [file S0950268825000317sup001.pdf]

Supplementary material

Annex 1. Roll-out of booster doses by study site ... 3

Table S1a. Date of COVID-19 vaccines roll-out by age group, number of booster doses, and study site 3

Table S1b. Date of 2022 autumn COVID-19 booster vaccination campaignm by age group and study site ... 4

Annex 2. Methodological details in the seven study sites ... 5

Table S2. Data sources used in the six study sites to extract the study variables ... 5

Table S3. Definition of covariates, categorisation and use in the model ... 7

Annex 3. Ethical statements for the seven study sites ... 11

Annex 4. Number of individuals, person-months and events included in the analysis ... 12

Table S4. Number of individuals, hospitalisations due to COVID-19, and person-months, according to two approaches. First methodological approach: relative vaccine effectiveness (VE) of the first, second and third booster dose, compared to complete primary vaccination without booster administered ≥24 weeks ago. Second methodological approach: annual (bivalent) vaccine effectiveness among individuals eligible for annual vaccine. VE estimates across each 8-week period in ≥80-year-olds, November 2022-July 2023. ... 12

Table S5. Number of individuals, hospitalisations due to COVID-19, and person-months, according to two approaches. First methodological approach: relative vaccine effectiveness (VE) of the first, second and third booster dose, compared to complete primary vaccination without booster administered ≥24 weeks ago. Second methodological approach: annual (bivalent) vaccine effectiveness among individuals eligible for annual vaccine. VE estimates across each 8-week period in 65–79 years, November 2022-July 2023. ... 13

Annex 5. Sample characteristics: proportion of person-months by vaccination status and covariates ... 14

Figure S1. Proportion of person-months (%) split by age and vaccination status, November 2022-July 2023. ... 14

Figure S2a. Proportion of person-months (%) split by sex and vaccination status, 80 years, November 2022-July 2023. ... 15

Figure S2b. Proportion of person-months (%) split by sex and vaccination status, 65–79 years, November 2022-July 2023. ... 16

Figure S3a. Proportion of person-months (%) split by site and vaccination status, 80 years, November 2022-July 2023. ... 17

Figure S3b. Proportion of person-months (%) split by site and vaccination status, 65–79 years, November 2022-July 2023. ... 18

Figure S4a. Proportion of person-months (%) split by comorbidities and vaccination status, 80 years, November 2022-July 2023. ... 19

Figure S4b. Proportion of person-months (%) split by comorbidities and vaccination status, 65–79 years, November 2022-July 2023. ... 20

Figure S5a. Proportion of person-months (%) split by country of birth and vaccination status, 80 years, November 2022-July 2023. ... 21

Figure S5b. Proportion of person-months (%) split by country of birth and vaccination status, 65–79 years, November 2022-July 2023. ... 22

Figure S6a. Proportion of person-months (%) split by citizenship and vaccination status, 80 years, November 2022-July 2023. ... 23

Figure S6b. Proportion of person-months (%) split by citizenship and vaccination status, 65–79 years, November 2022-July 2023. ... 24

Figure S7a. Proportion of person-months (%) split by number of booster doses received previously and vaccination status, 80 years, November 2022-July 2023. ... 25

Figure S7b. Proportion of person-months (%) split by number of booster doses received previously and vaccination status, 65–79 years, November 2022-July 2023. ... 26

Figure S8a. Proportion of person-months (%) split by vaccine product received and vaccination status, 80 years, November 2022-July 2023. 27
Figure S8b. Proportion of person-months (%) split by vaccine product received and vaccination status, 65–79 years, November 2022-July 2023. 28

Annex 6. Vaccine effectiveness against COVID-19 hospitalisation by time since vaccination 29

Table S6. Relative vaccine effectiveness (95% confidence intervals) in those aged ≥80 years against hospitalisation due to COVID-19 of the first, second and third booster dose, compared to complete primary vaccination without booster administered ≥24 weeks ago, by time since the booster, according to two approaches. First methodological approach: vaccine effectiveness (VE) of the first, second and third booster dose, compared to complete primary vaccination without booster administered ≥24 weeks ago. Second methodological approach: annual (bivalent) vaccine effectiveness among individuals eligible for annual vaccine. VE estimates for each 8-week overlapping study period, November 2022-July 2023. 29
Table S7. Vaccine effectiveness (95% confidence intervals) in those aged 65–79 years against hospitalisation due to COVID-19 of the first, second and third booster dose, compared to complete primary vaccination without booster ≥24 weeks ago, by time since the booster, according to two approaches. First methodological approach: vaccine effectiveness (VE) of the first, second and third booster dose, compared to complete primary vaccination without booster administered ≥24 weeks ago. Second methodological approach: annual (bivalent) vaccine effectiveness among individuals eligible for annual vaccine. VE estimates for each 8-week overlapping study period, November 2022-July 2023. 30

Annex 7. Vaccine effectiveness against COVID-19 mortality, overall...31

Table S8. Vaccine effectiveness (95% confidence intervals) in those aged ≥80 years against against COVID-19 related death according to two approaches. First methodological approach: vaccine effectiveness (VE) of the first, second and third booster dose, compared to complete primary vaccination without booster administered ≥24 weeks ago. Second methodological approach: annual (bivalent) vaccine effectiveness among individuals eligible for annual vaccine. VE estimates for each 8-week overlapping study period, November 2022-July 2023. 31
Table S9. Vaccine effectiveness (95% confidence intervals) in those aged 65 to 79 years against COVID-19 related death according to two approaches. First methodological approach: vaccine effectiveness (VE) of the first, second and third booster dose, compared to complete primary vaccination without booster administered ≥24 weeks ago. Second methodological approach: annual (bivalent) vaccine effectiveness among individuals eligible for annual vaccine. VE estimates or each 8-week overlapping study period, November 2022-July 2023. 32

Annex 8. Vaccine effectiveness against COVID-19 mortality, by time since vaccination 33

Table S10. Vaccine effectiveness (95% confidence intervals) in those aged ≥80 years against COVID-19 related death of the first, second and third booster dose, compared to complete primary vaccination without booster administered ≥24 weeks ago, according to two approaches. First methodological approach: vaccine effectiveness (VE) of the first, second and third booster dose, compared to complete primary vaccination without booster administered ≥24 weeks ago. Second methodological approach: annual (bivalent) vaccine effectiveness among individuals eligible for annual vaccine. VE estimates for each 8-week overlapping study period, November 2022-July 2023. 33
Table S11. Vaccine effectiveness (95% confidence intervals) in those aged 65–79 years against COVID-19 related death of the first, second and third booster dose, compared to complete primary vaccination without booster ≥24 weeks ago, according to two approaches. First methodological approach: vaccine effectiveness (VE) of the first, second and third booster dose, compared to complete primary vaccination without booster administered ≥24 weeks ago. Second methodological approach: annual (bivalent) vaccine effectiveness among individuals eligible for annual vaccine. VE estimates for each 8-week overlapping study period, November 2022-July 2023. 34

Annex 1. Roll-out of booster doses by study site

Table S1a. Date of COVID-19 vaccines roll-out by age group, number of booster doses, and study site

	≥80 years old				65-79 years old			
	Complete primary vaccination	Booster 1	Booster 2	Booster 3	Complete primary vaccination	Booster 1	Booster 2	Booster 3
Belgium	05.03.2021	22.09.2021	20.07.2022	12.09.2022	21.03.2021	22.09.2021	12.09.2022	-
Denmark	28.12.2020	18.10.2021	15.09.2022	-	15.03.2021	18.10.2021	01.10.2022	-
Italy	01.02. 2021	27.09.2021	08.04.2022	17.10.2022	07.04.2021	08.10.2021	11.07.2022	17.10.2022
Navarre (Spain)	02.03.2021	25.10.2021	10.10.2022	-	09.04.2021	25.10.2021	10.10.2022	-
Netherlands	04.02.2021	18.11.2021	26.02.2022	19.09.2022	19.04.2021	12.12.2021	26.03.2022	10.10.2022
Norway	31.01.2021	05.10.2021	01.07.2022	24.03.2023	07.03.2021	05.10. 2021	01.07.2022	-
Portugal	03.02.2021	11.10.2021	16.05.2022	06.09.2022	30.03.2021	11.10.2021	06.09.2022	-

Table S1b. Date of 2022 autumn COVID-19 booster vaccination campaign by age group and study site

	Age group	
	**≥80 years old**	**65-79 years old**
Belgium	12.09.2022	12.09.2022
Denmark	15.09.2022	01.10.2022
Italy	17.10.2022	17.10.2022
Navarre (Spain)	10.10.2022	10.10.2022
Netherlands	19.09.2022	10.10.2022
Norway	01.07.2022	01.07.2022
Portugal	06.09.2022	06.09.2022

Annex 2. Methodological details in the seven study sites

Table S2. Data sources used in the six study sites to extract the study variables

Type of variables	Study variable	Study site						
		**Belgium**	**Denmark**	**Italy**	**Navarre (Spain)**	**The Netherlands**	**Norway***	**Portugal**
**Outcomes**	**Hospital admission due to COVID-19**	Clinical Hospital Survey database	Danish National Patient Register (DNPR)	National Integrated COVID-19 Surveillance Databases	Enhanced COVID surveillance with individual revision of events	NICE COVID-19 registration	Norwegian Intensive Care and Pandemic Registry (NIPaR)	National Hospital Discharge database (BIMH)
	**Death due to COVID-19**	Not applicable	MiBA and Danish Civil Registration system (CPR)	National Integrated COVID-19 Surveillance Databases	Administrative database of deaths and individual revision of events	NA	Norwegian Death Registry (DÅR)	National Death Registry (SICO) and National Health Service User database (NHSU): a Cause of death is from SICO, death status and date of death from NHSU.
**Exposures**	**Vaccination status**	National vaccine registry (VACCINNET)	Danish Vaccination Registry (DVR)	National Vaccination Registry	Vaccination register	COVID-19 vaccination registry (CIMS)	The National Immunisation Register (SYSVAK)	The National Vaccination Register (VACINAS)
	**Number of previous booster doses****	National vaccine registry (VACCINNET)	Danish Vaccination Registry (DVR)	National Vaccination Registry	Vaccination register	COVID-19 vaccination registry (CIMS)	The National Immunisation Register (SYSVAK)	The National Vaccination Register (VACINAS)
**Variables for adjustment or stratification**	**Age**	The national population register	CPR	National Vaccination Registry	Administrative database	The national population register	The National Population Register (Folkeregisteret)	National Health Service User database (NHSU)
	**Sex**	national population register	CPR	National Vaccination Registry	Administrative database	The national population register	The National Population Register (Folkeregisteret)	National Health Service User database (NHSU)
	**Region**	Province of residence: national population register	CPR	Region where vaccination took place: National Vaccination Registry	Not applicable	NA	County of residence at end of study period: The National Population Register (Folkeregisteret)	Region of residence: National Health Service User database (NHSU)

	**Comorbidities**	Intermutualistic Agency database	DNPR	National Vaccination Registry	Primary Care Information System	Specialized health care utilization combined with prescribed medication register, both from year 2020	Risk groups / Comorbidities: Based on Norwegian Patient Registry (NPR)	Primary Care Information System (SIM@SNS).
	**Previous infection*** **	COVID-19 Laboratory test results database from Healthdata.be register	MiBA	National Integrated COVID-19 Surveillance Databases	Previous infections are excluded, pendent of a separate analysis	NA	The Surveillance System for Infectious Diseases (MSIS)	National Information System for Epidemiologic Surveillance (BI-SINAVE)
	**Others specific to the study site**	Household income (according to tax records) categorized as low (lowest 40%), mid (middle 30%), and high (highest 30%): STATBEL database	Not applicable	Country of birth: National Vaccination Registry	Country of birth and high functional dependence: Administrative database	Months of death and emigration from population registry, LTCF residency from Central Administration Office	1. Conditions of living – Crowding: Statistics Norway (SSB). Most recent data from 2019 – separate level for missing data 2. *County of birth:* Folkeregisteret	1. Number of tests for SARS-CoV-2 in 2020-2022: BI-SINAVE 2. Conditions of living – Deprivation at municipality level: Most recent data from 2011 3. Other vaccines uptake: VACINAS

***All data in Norway was integrated in the emergency preparedness register for COVID-19 (Beredt C19), https://www.fhi.no/en/id/infectious-diseases/coronavirus/emergency-preparedness-register-for-covid-19/

** Considered only in the second methodological approach

*** Considered only in the first methodological approach

Table S3. Definition of variables, categorisation and use in the model

Variable	Definition, categorisation, use in the model							
	**Belgium**	**Denmark**	**Italy**	**Luxembourg**	**The Netherlands**	**Navarre (Spain)**	**Norway**	**Portugal**
**Age**	Age in years at the end of the year in which the study period begins.<br>For adjustment: 5-year age groups.	5-17, 18-49, 50-64, 65-79, ≥80, adjusted in categories: 5-9, 10-14, 15-17, 18-24 and then 5-year categories until the final category, 90+ years	Age at the start of study period (for adjustment: 5-year age groups up to 90-94 years and then grouping ≥95 years))	Age at the start of follow up, 5-year categories	Age at the start of study period (5-year categories)	Age at the start of study period (5-year categories)	Age at end of 2022 (birth cohorts) (For adjustment: 5-year age groups)	Age at the start of study period (5-year categories)
**Comorbidities**	**Medium risk:**<br>At least one comorbidity which increases the risk for severe COVID-19 infection and not being immunocompromised (medium risk):<br>- *Received chemotherapy/ radiotherapy against cancer*<br>- *Received multidisciplinary oncologic consult*<br>- *Cardiovascular illness – general*<br>- *Cardiovascular illness-specifically a heart disease*<br>- *Alzheimer*<br>- *Asthma*<br>- *Haemophilia*<br>- *Disease of Crohn, Colitis Ulcerosa, Psoriatrische arthritis, Reumatoid arthritis*<br>- *Chronic obstructive pulmonary disease*<br>- *Diabetes with cardiovascular compilations*<br>- *Diabetes Mellitus with insulin treatment*<br>- *Epilepsy and neuropathic pain*<br>- *Chronic hepatitis type B or C*<br>- *Kidney failure*<br>- *Cystic fibrosis*<br>- *Exocrine pancreatic disease*<br>- *Disease of Parkinson*<br>- *Psoriasis*	**Medium risk:**<br>Other, including:<br>- *Diabetes*<br>- *Obesity*<br>- *Cancer*<br>- *Neurological Disease*<br>- *Kidney disease*<br>- *Haematological cancers*<br>- *Heart disease*<br>- *Chronic respiratory disease*<br>- *Liver disease (incl. alcohol lever)*<br>- *Endocrine Disease*<br>- *Hematological Disease*<br>- *Coagulation Disease*<br>- *Innate Diseases*<br>- *TB*<br>- *Missing a lung*<br>- *Missing a kidney*<br><br>**High risk:**<br>Immunocompromised, including:<br>- *HIV*<br>- *Immunological disease*<br>- *Radiation therapy*<br>- *Organ transplanted*	**Medium risk:**<br>Other comorbidities, including:<br>- *Respiratory diseases requiring oxygen therapy, idiopathic pulmonary fibrosis*<br>- *Advanced heart failure (Classes III-IV NYHA) and post cardiogenic shock patients*<br>- *Amyotrophic lateral sclerosis and other motor neuron disorders, multiple sclerosis, muscular dystrophy, infantile cerebral palsy, myasthenia gravis, dysimmune neuropathies*<br>- *Type 1 diabetes, Type 2 diabetes with complications or requiring combination therapy (with at least two anti-diabetes drugs)*<br>- *Addison's disease*<br>- *Panhypopituitarism*<br>- *Cystic fibrosis*<br>- *-Cirrhosis of the liver*<br>- *Intracerebral ischemic or hemorrhagic event that has led to impaired neurological and cognitive autonomy*<br>- *Individuals who have had a stroke on 2020 or later ranked as level 3 or higher*<br>- *Thalassemia major*<br>- *Sickle cell anemia*<br>- *Other severe anemias*<br>- *Down syndrome*<br>- *Body Mass Index >35*	Not included	**Medium risk:**<br>eligibility for influenza vaccination based on comorbid conditions.<br><br>**High risk**: is defined as comorbid conditions based on increased risk of severe covid. Methods, data and codes used are extensively described in the article by de Gier et al. [16].	**Medium risk:**<br>Other major chronic conditions<br>- *Diabetes*<br>- *Severe Obesity*<br>- *Ictus*<br>- *Dementia*<br>- *Kidney disease*<br>- *Non-haematologic al cancers*<br>- *Heart disease*<br>- *Chronic respiratory disease*<br>- *Liver disease*<br>- *Rheumatic arthritis*<br><br>**High risk:**<br>Immunocompr omised including:<br>- HIV-infection<br>- Organ transplanted<br>- Haematologic al cancers<br>- Congenital immunodefici ency	**Medium risk:**<br>- *Chronic liver disease or significant hepatic impairment*<br>- *Immunosuppressive therapy*<br>- *Diabetes*<br>- *Chronic lung disease including cystic fibrosis and severe asthma which have required the use of high dose inhaled or oral steroids within the past year*<br>- *Obesity with a body mass index (BMI) of ≥35 kg/m2*<br>- *Dementia*<br>- *Chronic heart and vascular disease (with the exception of high blood pressure) and stroke*<br><br>**High risk:**<br>- *Organ transplant*<br>- *Immunodeficiency*<br>- *Haematological cancer in the last five years*<br>- *Other active cancers*<br>- *Neurological or neuromuscular diseases that cause*	Number of comorbidities (0, 1, 2, 3, 4, 5+)<br><br>*Considered comorbidities include: anemia, asthma, cancer, cardiac disease, dementia, diabetes, hypertension, HIV, liver disease, neuromuscular disease, obesity, pulmonary disease, renal disease, rheumatologic disease, stroke, tuberculosis*

	- *Psychosis occurring with people older than 70 years*<br>- *Psychosis occurring with people of 70 year or younger.*<br>- *Multiple sclerosis*<br>- *Thrombosis while treated with antithrombotic medicines*<br>- *Thyroid disorder*<br>- *HIV*<br><br>**High risk:**<br>- Immunocompromised (high risk):<br>- *Disease of Crohn, Colitis Ulcerosa, Psoriatrische arthritis, Reumatoid arthritis*<br>- *Kidney failure*<br>- *Cystic fibrosis*<br>- *Psoriasis*<br>- *Multiple sclerosis*<br>- *Organ transplantation*<br>- *Received chemotherapy/radiotherapy against cancer*<br>- *Received multidisciplinary oncologic consult*		- *Severely disabled persons pursuant to law 104/1992 art. 3 paragraph 3*<br>- *Chronic Alcohol Misuse*<br>- *Functional or anatomic asplenia*<br>- *COPD*<br>- *Chemotherapy or Radiotherapy*<br>- *Coagulopathies*<br>- *Diabetes Mellitus and other endocrinopathies*<br>- *Patients in hemodialysis or with chronic kidney diseases expected to start dialysis*<br>- *Hemoglobinopathy such as sickle cell anemia or thalassemia*<br>- *Chronic Liver Disease*<br>- *Cochlear implant*<br>- *Chronic Kidney Disease*<br>- *Chronic eczema or psoriasis*<br>- *Diseases associated with a high risk of aspiration pneumonia*<br>- *Chronic Cardiovascular Disease*<br>- *Chronic Respiratory Disease*<br>- *Motor neuron diseases*<br>- *Chronic inflammatory diseases and malabsorption syndromes*<br>- *Blood cancers (leukemia, lymphoma and myeloma)*<br>- *Solid tumors*<br>- *Obesity (Body Mass Index 30-35)*<br>- *Bone marrow transplant*<br>- *Drug Misuse*<br>- *Solid organ transplant*<br>- *Patients with CSF leak from trauma or intervention*<br>- *Patients going to start immunosuppressive treatment*<br>- *Metabolic diseases*<br>- *Hematopoietic diseases*<br>- *Pathologies that require important surgical interventions*<br>- *Neurological diseases*<br>- *Cerebrovascular diseases*<br>- *Down Syndrome*<br>- *Disabilities (physical, sensorial, learning or psychic)*<br><br>**High risk:**			- Asplenia	*impaired cough or lung function (e.g., ALS and cerebral palsy)*<br>- *Chronic kidney disease, or significant renal impairment.*	

			Immunocompromised, including:<br>- *Immunocompromised defects of the complement system*<br>- *Other specified disorders involving the immune mechanism*<br>- *Deficiency or dysfunction of a single component (C1-C9)*<br>- *Deficiency of cell-mediated immunity*<br>- *Deficiency of humoral immunity*<br>- *Human immunodeficiency virus [HIV] disease, Human immunodeficiency virus, type 2 [HIV-2], Asymptomatic human immunodeficiency virus [HIV] infection status*<br>- *Disorders involving the immune mechanism*<br>- *Congenital and acquired disorders with poor antibody production*<br>- *Drug-induced immunosuppression*<br>-					
**Country of residence / country of birth / nationality**	Not included	Not included	Country of birth: born in Italy; born in other countries	Country of residence = administrative address in Luxembourg (as of September 2021)<br>Country of birth = Luxembourg / Other<br>Nationality = Citizenship Luxembourg / Other	Not included	Country of birth	Registered as living in Norway.<br>Country of birth: Norway / not Norway / unknown	Not available
**Deprivation index or similar**	Household income: low (lowest 40%)-medium (middle 30%)-high (highest 30%)	Not included	Not included	Not included	Not included	High functional dependence	Crowded conditions: if the number of rooms is lower than the number of residents or one resident lives in one room, and the number of square metres (P-area) is below 25 sq. m. per person. If the number	European deprivation index quintile Q1 (least deprived) to Q5 (most deprived)

							of rooms or the P-area is not specified, a household was regarded as crowded if one of these criteria is met (incomplete and slightly outdated data)	
**Geographic level**	Province of residence	Adjustment for residency in the 5 geographical regions of Denmark (EU NUTS-2 regions)	19 regions and 2 autonomous provinces of Italy where vaccination took place	Canton	Not included	Only one province is included	County of residence	Region of residence (North, Center, Lisbon and Tagus Valey, Alentejo, Algarve)
**Other vaccines uptake**	Not included	Not included	Not included	Not included	Not included	Not included	Not included	Vaccination against influenza, PCV7, PCV10, PCV13 or PPV23 in the last 3 years
**Number of COVID-19 tests in 2020-2022**	Not included	Positive RT-PCR test for SARS-CoV-2	Not included	Not included	Not included	Not included	Not included	0, 1, 2, 3, 4-9, 10+

Annex 3. Ethical statements for the seven study sites

All study sites participating in this study conformed with their respective national and EU ethical and data protection requirements. Ethical statements for each of the participating study sites:

Belgium: Data linkage and collection within the data-warehouse have been approved by the information security committee. The study was conducted in accordance with the Declaration of Helsinki. Ethical approval was granted for the gathering of data from hospitalized patients by the Committee for Medical Ethics from the Ghent University Hospital (reference number BC-07507) and authorization for possible individual data linkage using the national register number from the Information Security Committee (ISC) Social Security and Health (reference number IVC/KSZG/20/384). Linkage of hospitalized patient data to vaccination and testing within the LINK-VACC project was approved by the Medical Ethics Committee UZ Brussels–VUB on 3 February 2021 (reference number 2020/523), and authorization from the ISC Social Security and Health (reference number IVC/KSZG/21/034).

Denmark: We used only administrative register data for the study. According to Danish law, ethics approval is exempt for such research, and the Danish Data Protection Agency, which is dedicated ethics and legal oversight body, thus waives ethical approval for our study of administrative register data when no individual contact of participants is necessary, and only aggregate results are included as findings. The study is, therefore, fully compliant with all legal and ethical requirements, and there are no further processes available regarding such studies.

Navarre (Spain): The study was approved by Navarre’s Ethical Committee for Clinical Research, which waived the requirement of obtaining informed consent.

Norway: Ethical approval was granted by Regional Committees for Medical and Health Research Ethics (REC) Southeast (reference number 122745). The Norwegian Institute of Public Health has performed a Data Protection Impact Assessment (DPIA) for Beredt C19.

Portugal: The study received approval from the Ethical Committee and the Data Protection Officer of the Instituto Nacional de Saúde Doutor Ricardo Jorge. Given that data was irreversibly anonymised, the need for the participants’ informed consent was waived by the Ethical Committee.

Netherlands: We performed a Data Protection Impact Assessment to identify potential protection and privacy risks and measures to mitigate or manage these risks. Based on this document the study has been assessed by the Central Privacy Team of the National Institute of Public Health after consultation with the Data Protection Officer from the Ministry of Health. Final approval was achieved from the Head of the Centre for Infectious Disease Epidemiology and Surveillance. Ethical approval and patients consents were not required although necessary measures have been taken to ensure patients’ privacy.

Italy: This study, based on routinely collected data, was not submitted for approval to an ethical committee because the dissemination of COVID-19 surveillance data was authorized by the Italian law N. 52 of 19 May 2022, following the law decree N. 24 of 24 March 2022 (Article n. 13). Based on the same acts, the information on COVID-19 vaccination was retrieved by the Italian National Institute of Health using data from the National Immunisation Information System of the Italian Ministry of Health. Because of the retrospective design and the large size of the population under study, in accordance with the Authorization n. 9 released by the Italian data protection authority on 15 December 2016, the individual informed consent was not requested for the conduction of this study.

Annex 4. Number of individuals, person-months and events included in the analysis

Table S4. Number of individuals, hospitalisations due to COVID-19, and person-months, according to two approaches. First methodological approach: relative vaccine effectiveness (VE) of the first, second and third booster dose, compared to complete primary vaccination without booster administered ≥24 weeks ago. Second methodological approach: annual (bivalent) vaccine effectiveness among individuals eligible for annual vaccine. VE estimates across each 8-week period in ≥80-year-olds, November 2022-July 2023.

Study period	First methodological approach								Second methodological approach			
	Primary vaccination ≥24 weeks ago without a booster		Complete primary vaccination + first booster dose		Complete primary vaccination + two booster doses		Complete primary vaccination + three booster doses		Eligible but did not receive the seasonal booster dose		Received the seasonal booster dose	
	N	Events/ person-month	N	Events/ person-month	N	Events/ person-month	N	Events/ person-month	N	Events/ person-month	N	Events/ person-month
Nov'22 -Dec'22	474,560	1,005/ 845,775	2,554,396	6750/ 4,276,515	2,589,931	5624/ 4,212,750	1,036,062	762/ 1,685,992	2,845,017	7,977/ 4,772,621	1,899,307	2,039/ 3,120,972
Dec'22- Jan'23	464,426	871/ 835,569	2,327,906	5426/ 4,072,376	2,538,978	4987/ 4,354,552	1,114,830	1,016/ 1,902,532	2,565,749	6,431/ 4,509,058	1,965,396	2,499/ 3,452,680
Jan'23- Feb'23	461,755	460/ 835577	2,251,506	2835/ 4,020,140	2,570,521	2748/ 4,565,112	1,173,244	1,016/ 2,078,505	2,461,440	3,330/ 4,407,652	2,044,885	1,862/ 3,671,236
Feb'23- Mar'23	459,347	355/ 833,012	2,197,866	2,158/ 3,982,995	2,535,418	2,579/ 4,584,882	1,185,525	1,450/ 2,132,358	2,368,548	2,604/ 4,294,268	1,955,211	2,364/ 3,552,464
Mar'23 -Apr'23	458,333	383/ 832,028	2,186,666	2,212/ 3,977,836	2,517,378	2,864/ 4,584,096	1,185,721	1,305/ 2,152,304	2,366,041	2,623/ 4,302,226	2,030,076	2,471/ 3,690,798
Apr'23- May'23	457,664	302/ 831,339	2,182,943	1,756/ 3,974,305	2,493,103	2,234/ 4,520,088	1,227,272	697/ 2,188,324	2,345,480	1,978/ 4,267,127	2,017,950	1,591/ 3,638,582
May'23 -Jun'23	454,035	137/ 825,216	2,072,663	847/ 3,769,324	1,982,081	941/ 3,559,457	984,723	322/ 1,766,726	2,327,429	1,007/ 4,237,216	1,980,263	850/ 3,597,574
Jun'23- Jul'23	456,016	79/ 829,390	2,076,542	419/ 3,780,071	2,193,302	490/ 3,996,201	965,713	179/ 1,748,257	2,311,164	508/ 4,209,484	1,961,016	433/ 3,580,608

Table S5. Number of individuals, hospitalisations due to COVID-19, and person-months, according to two approaches. First methodological approach: relative vaccine effectiveness (VE) of the first, second and third booster dose, compared to complete primary vaccination without booster administered ≥24 weeks ago. Second methodological approach: annual (bivalent) vaccine effectiveness among individuals eligible for annual vaccine. VE estimates across each 8-week period in 65–79 years, November 2022-July 2023.

Study period	First methodological approach								Second methodological approach			
	Primary vaccination ≥24 weeks ago without a booster		Complete primary vaccination + first booster dose		Complete primary vaccination + two booster doses		Complete primary vaccination + three booster doses		Eligible but did not receive the seasonal booster dose		Received the seasonal booster dose	
	N	Events/ person-month	N	Events/ person-month	N	Events/ person-month	N	Events/ person-month	N	Events/ person-month	N	Events/ person-month
Nov’22 -Dec’22	1,151,423	858/ 2,036,321	7,703,497	5,273/ 12,678,134	6,227,487	2,835/ 10,020,798	1,271,770	414/ 2,047,012	7,939,539	6,294/13,083,591	5,764,888	1,646/ 9,323,103
Dec’22-Jan’23	1,113,290	741/ 2,009,923	6,838,660	4,202/ 11,987,172	6,289,874	2,712/ 10,973,780	1,353,163	547/ 2,304,388	6,992,996	5,085/12,322,733	5,989,503	2,104/10,557,355
Jan’23-Feb’23	1,114,463	476/ 2,026,339	6,587,984	2,297/ 11,801,443	6,417,000	1,687/ 11,479,756	1,408,441	534/ 2,457,090	6,728,828	2,777/12,078,335	6,139,831	1,547/11,069,362
Feb’23-Mar’23	1,112,174	399/ 2,030,489	6,429,926	1,969/ 11,704,199	6,364,831	1,752/ 11,609,407	1,422,228	781/ 2,539,261	6,390,329	2,401/11,645,878	5,519,127	1,919/10,075,102
Mar’23 -Apr’23	1,116,291	368/ 2,039,725	6,402,563	1,889/ 11,703,573	6,348,105	1,863/ 11,641,729	1,426,943	697/ 2,567,173	6,521,587	2,313/11,921,418	6,156,940	2,101/11,260,714
Apr’23-May’23	1,118,440	255/ 2,043,974	6,373,563	1,515/ 11,665,286	6,266,592	1,420/ 11,476,071	1,424,150	335/ 2,569,717	6,502,317	1,781/11,898,021	6,145,841	1,380/11,200,112
May’23 -Jun’23	1,107,982	128/ 2,025,876	6,283,390	799/ 11,499,953	5,461,271	602/ 9,960,057	1,423,789	152/ 2,571,486	6,490,934	971/11,881,494	6,091,374	736/11,143,367
Jun’23-Jul’23	1,108,402	61/ 2,027,847	5,974,550	327/ 10,929,614	4,315,180	295/ 7,881,074	1,365,863	64/ 2,469,075	6,481,610	428/11,868,417	6,064,916	349/11,122,229

Annex 5. Sample characteristics: proportion of person-months by vaccination status and covariates

Figure S1. Proportion of person-months (%) split by age and vaccination status, November 2022-July 2023.

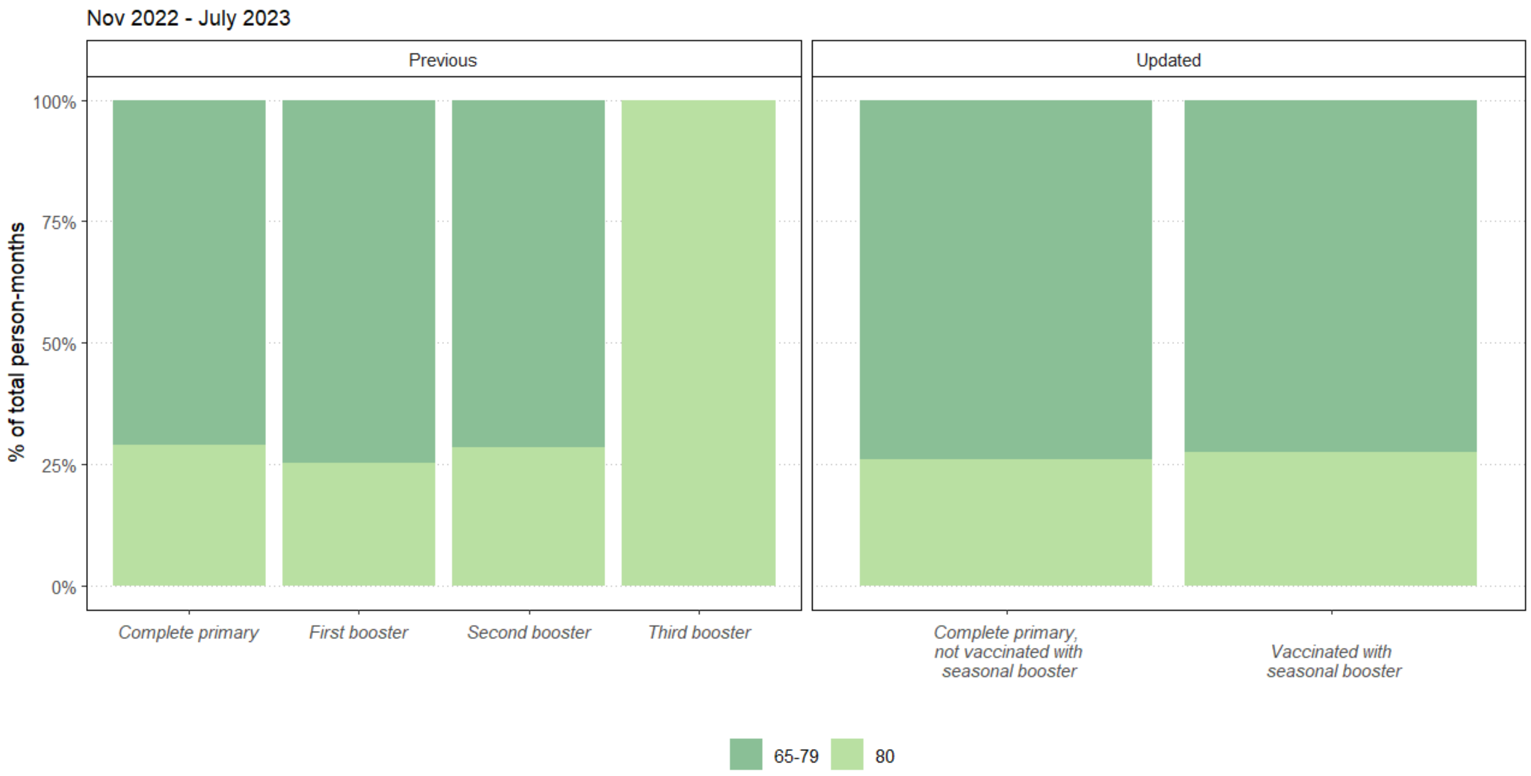

Figure S2a. Proportion of person-months (%) split by sex and vaccination status, 80 years, November 2022-July 2023.

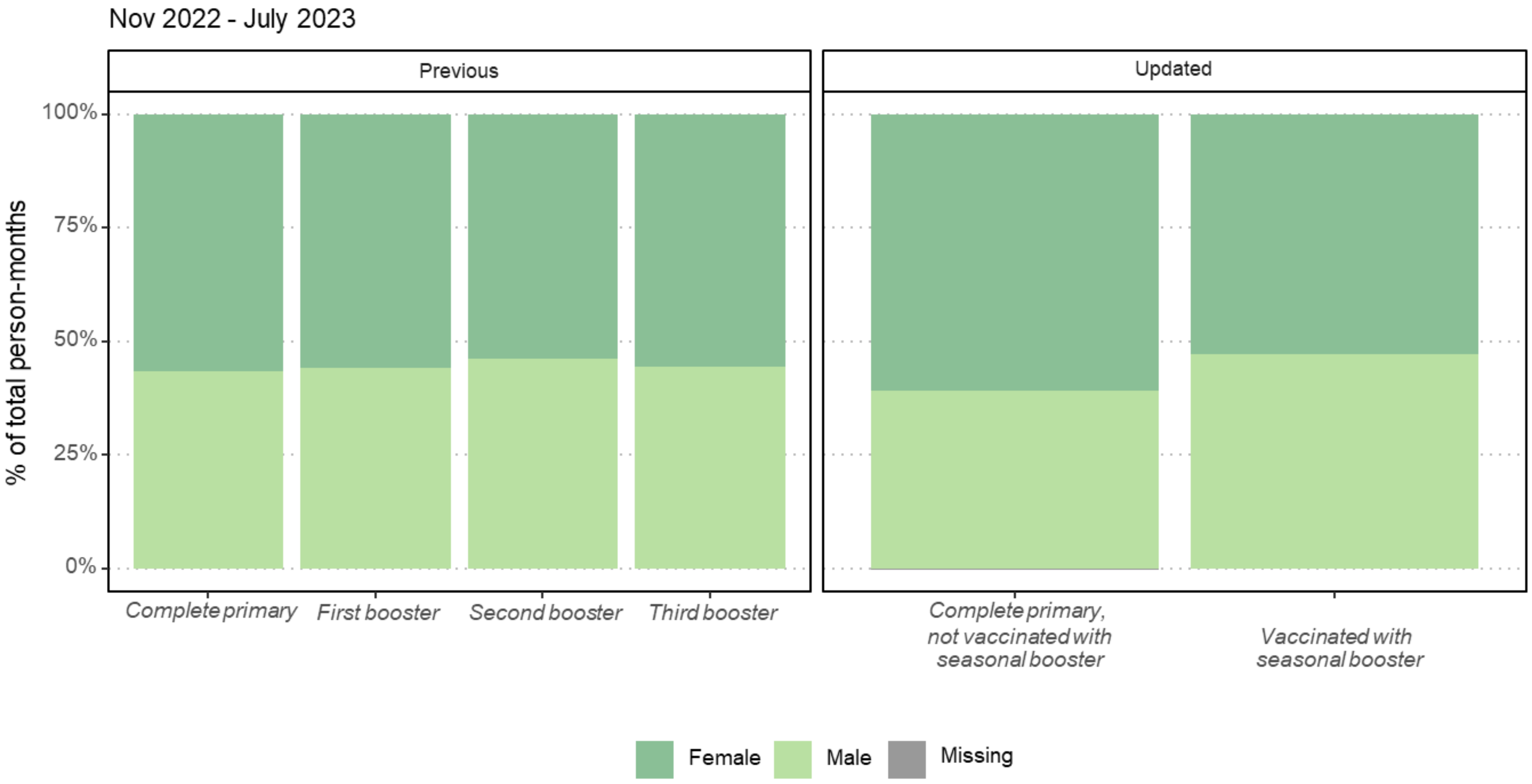

Figure S2b. Proportion of person-months (%) split by sex and vaccination status, 65–79 years, November 2022-July 2023.

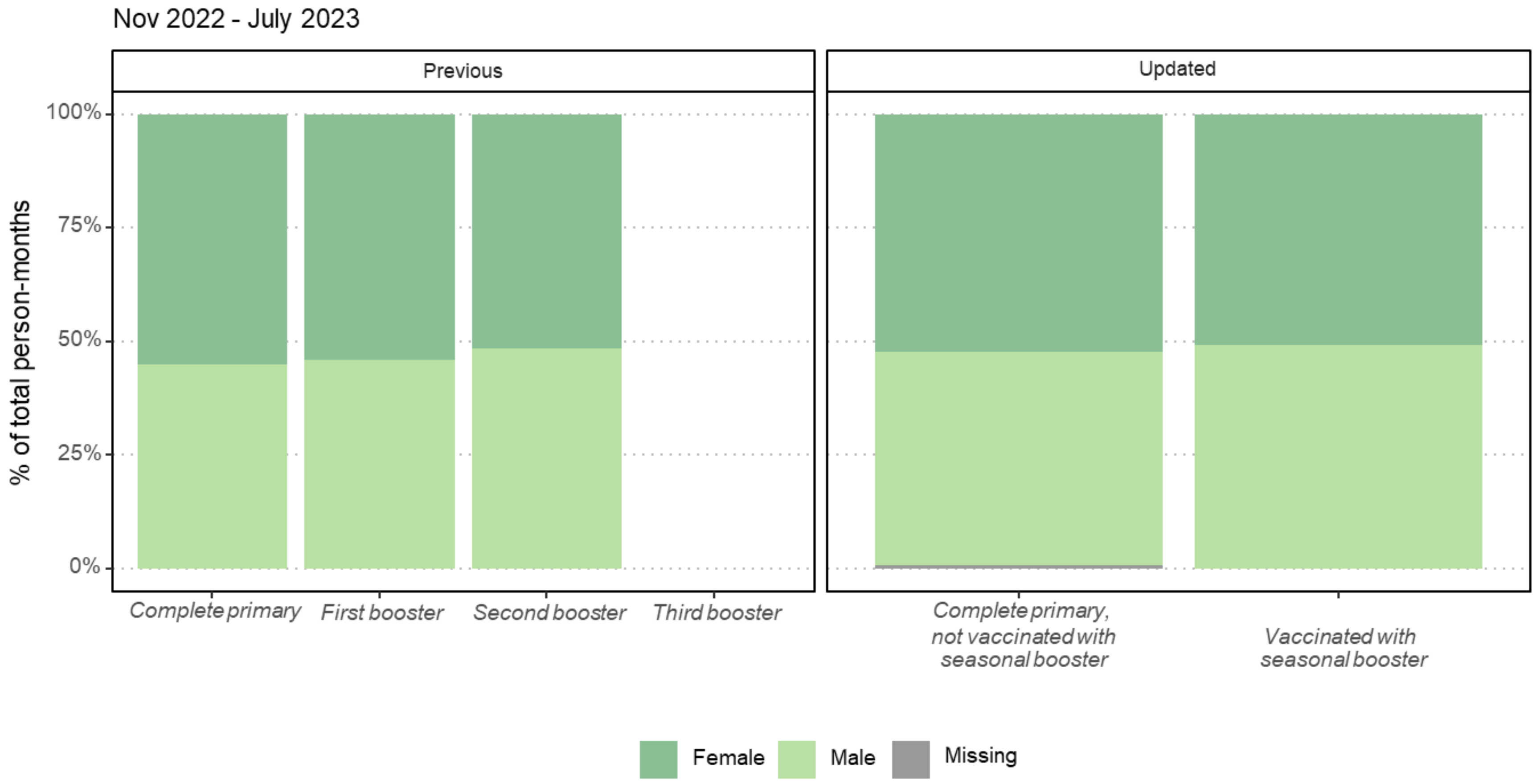

Figure S3a. Proportion of person-months (%) split by site and vaccination status, 80 years, November 2022-July 2023.

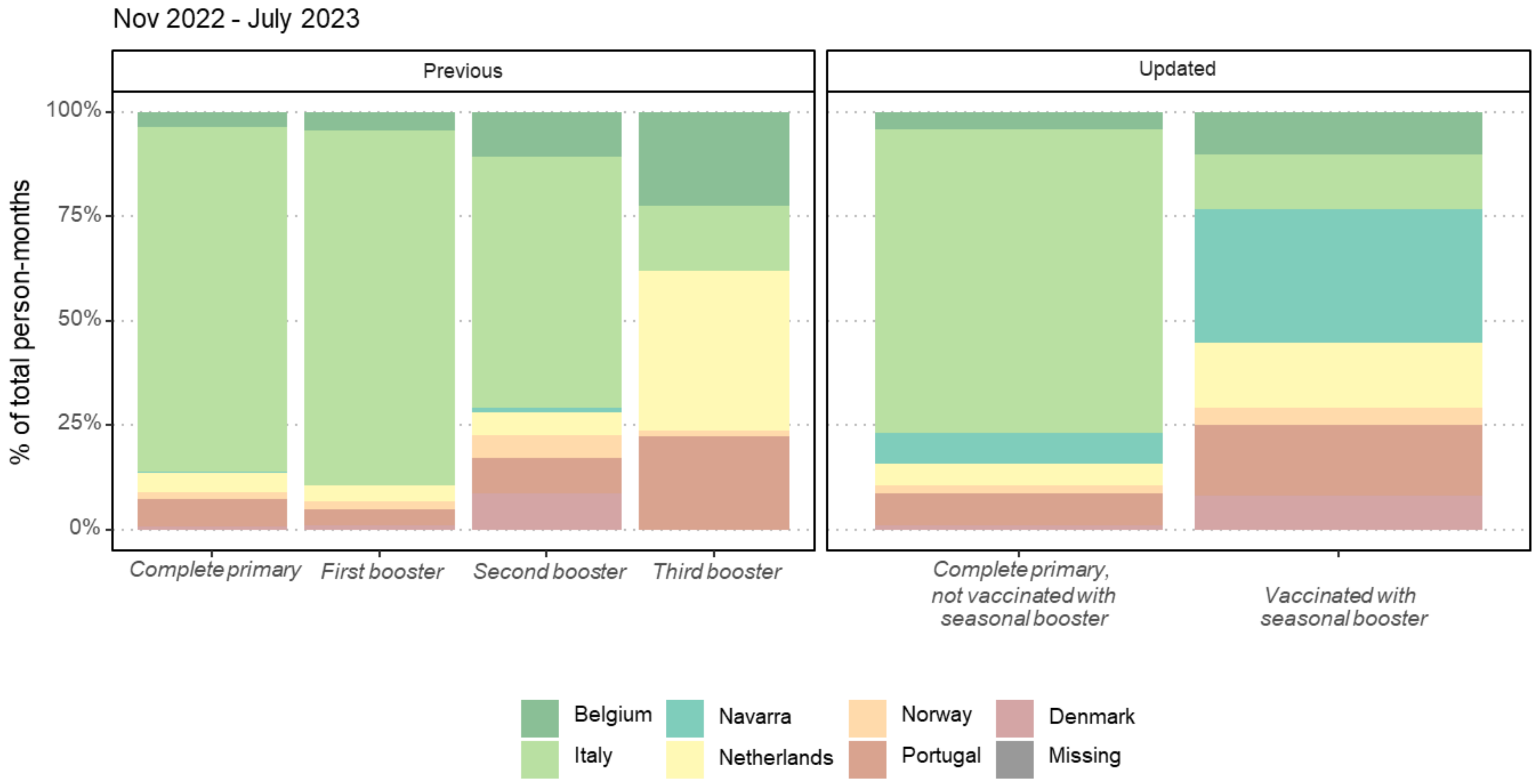

Figure S3b. Proportion of person-months (%) split by site and vaccination status, 65–79 years, November 2022-July 2023.

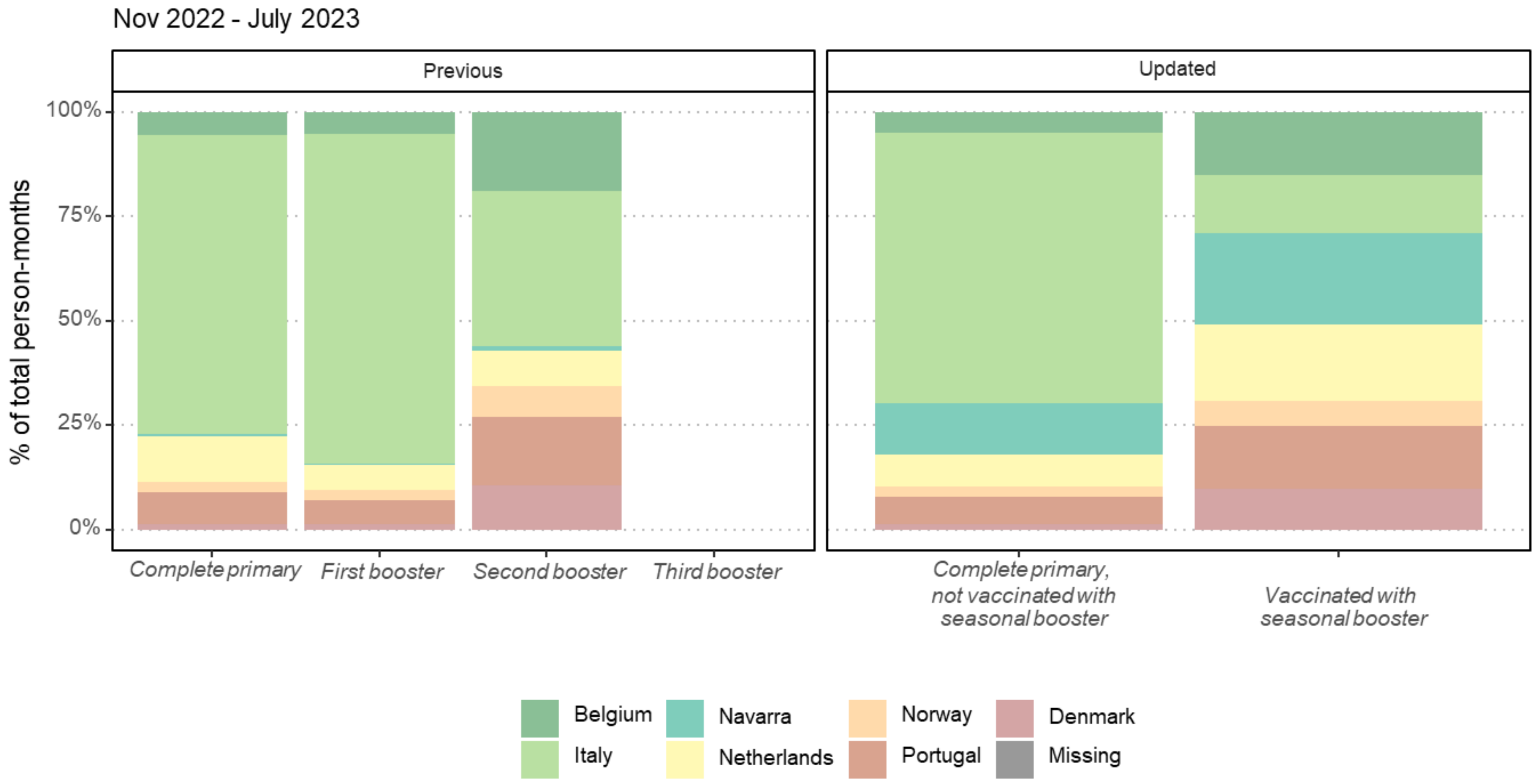

Figure S4a. Proportion of person-months (%) split by comorbidities and vaccination status, 80 years, November 2022-July 2023.

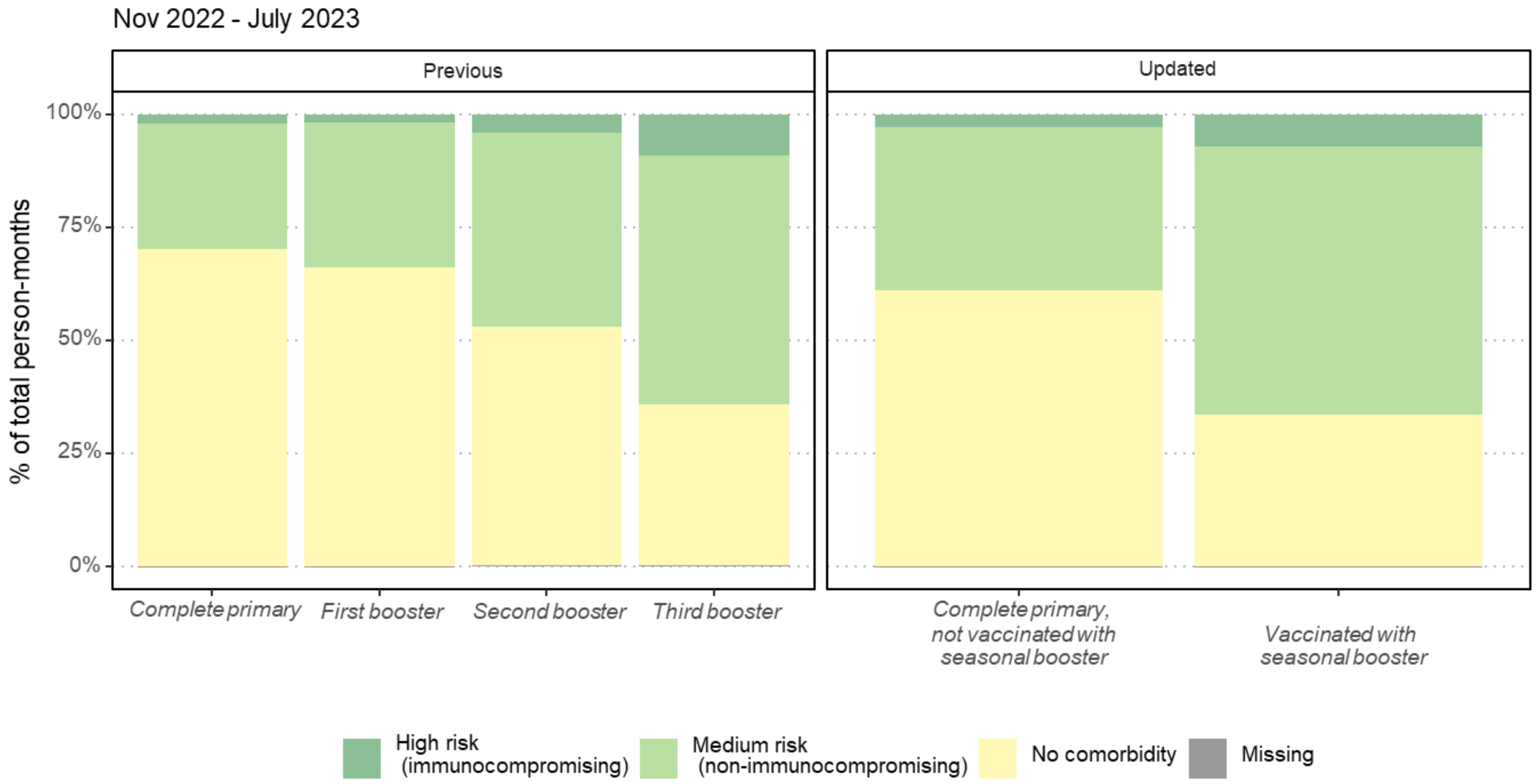

Figure S4b. Proportion of person-months (%) split by comorbidities and vaccination status, 65–79 years, November 2022-July 2023.

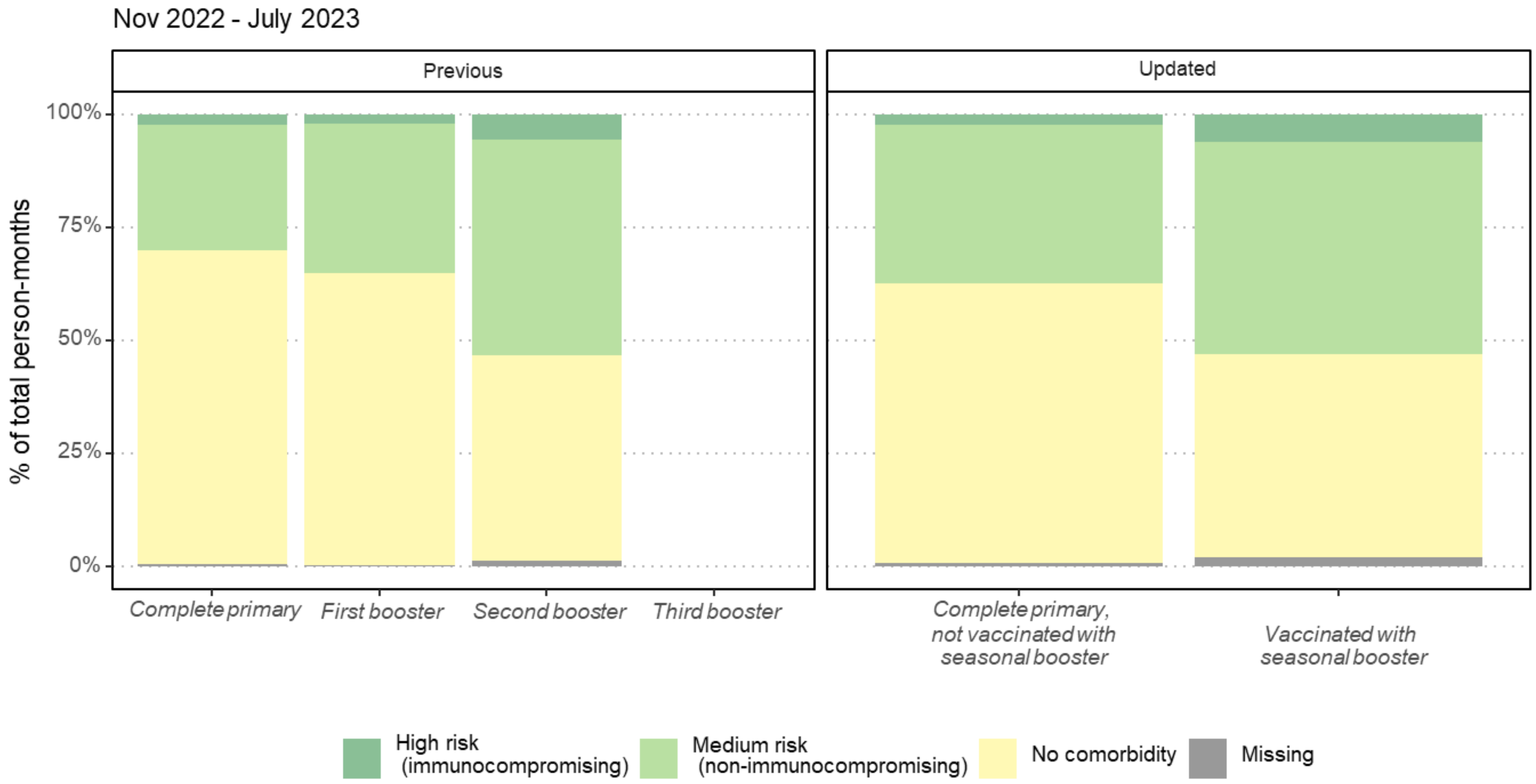

Figure S5a. Proportion of person-months (%) split by country of birth and vaccination status, 80 years, November 2022-July 2023.

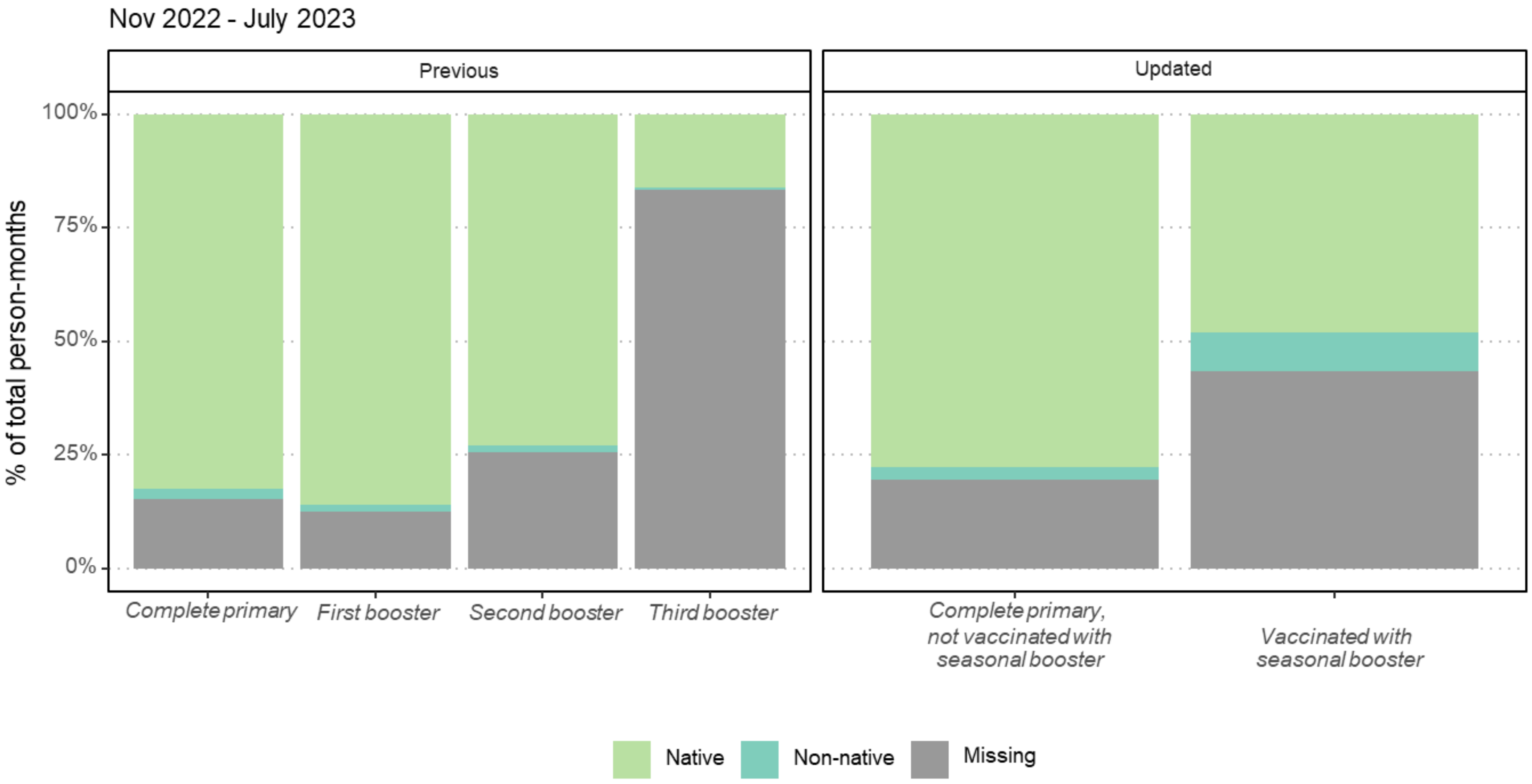

Figure S5b. Proportion of person-months (%) split by country of birth and vaccination status, 65–79 years, November 2022-July 2023.

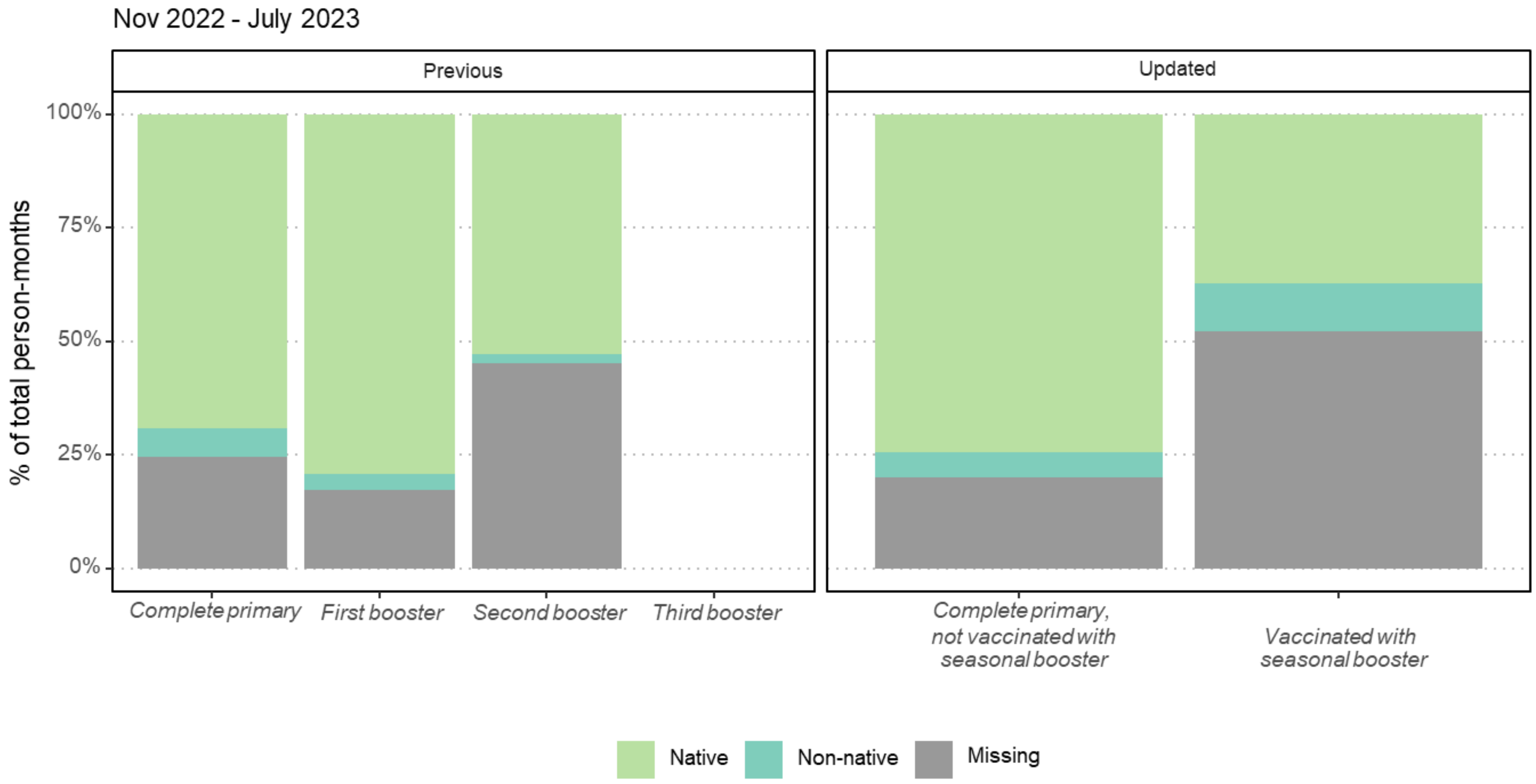

Figure S6a. Proportion of person-months (%) split by citizenship and vaccination status, 80 years, November 2022-July 2023.

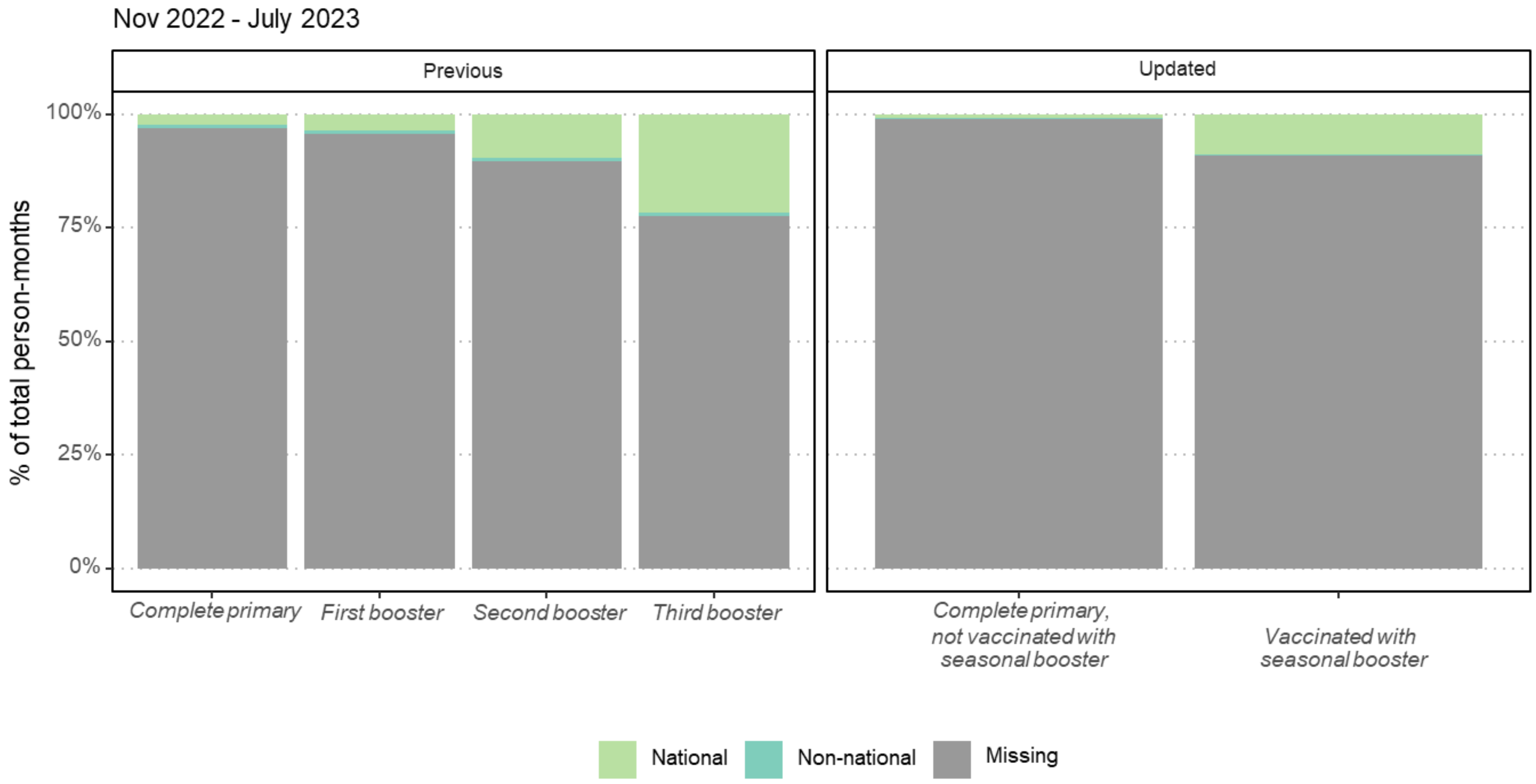

Figure S6b. Proportion of person-months (%) split by citizenship and vaccination status, 65–79 years, November 2022-July 2023.

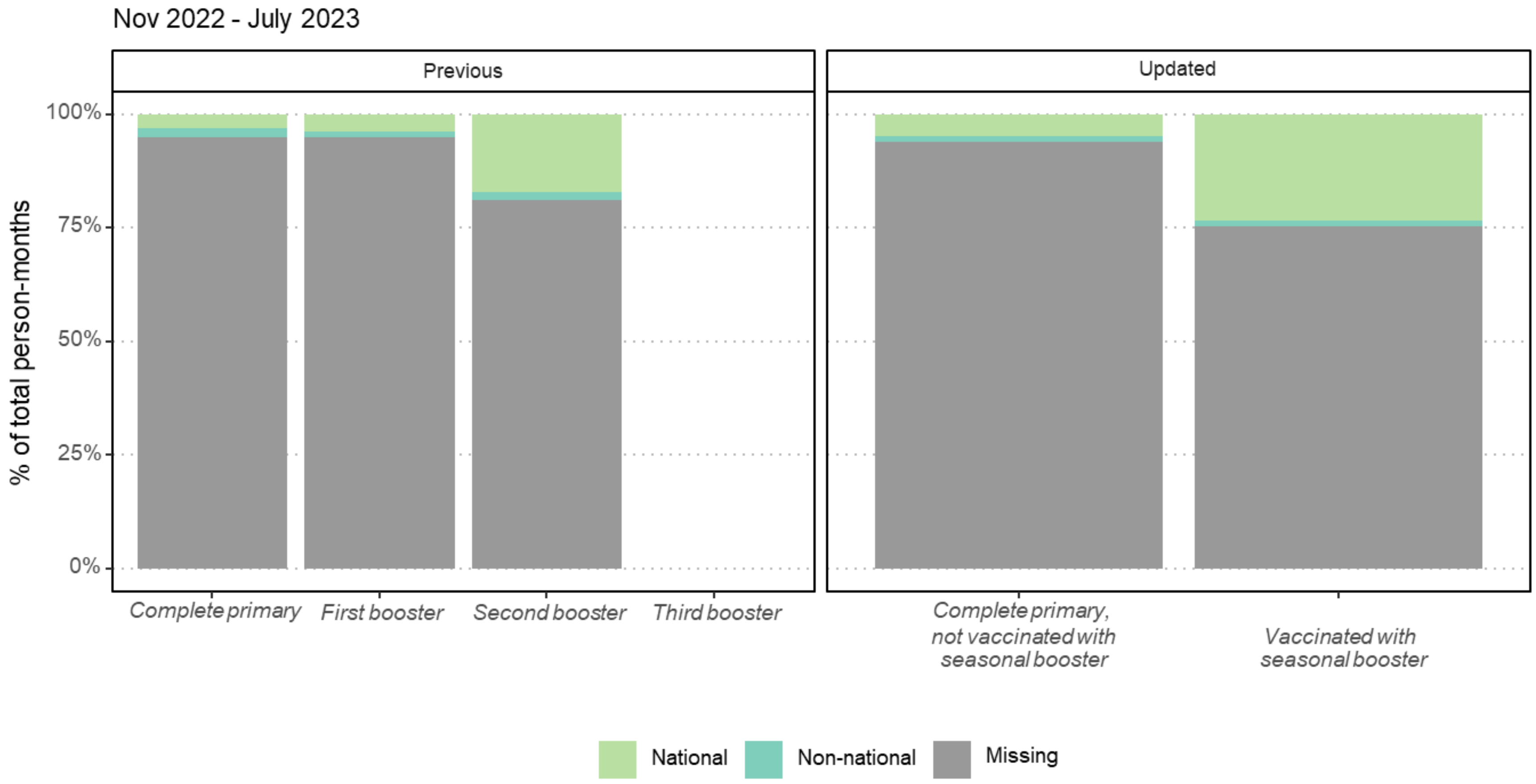

Figure S7a. Proportion of person-months (%) split by number of booster doses received previously and vaccination status, 80 years, November 2022-July 2023.

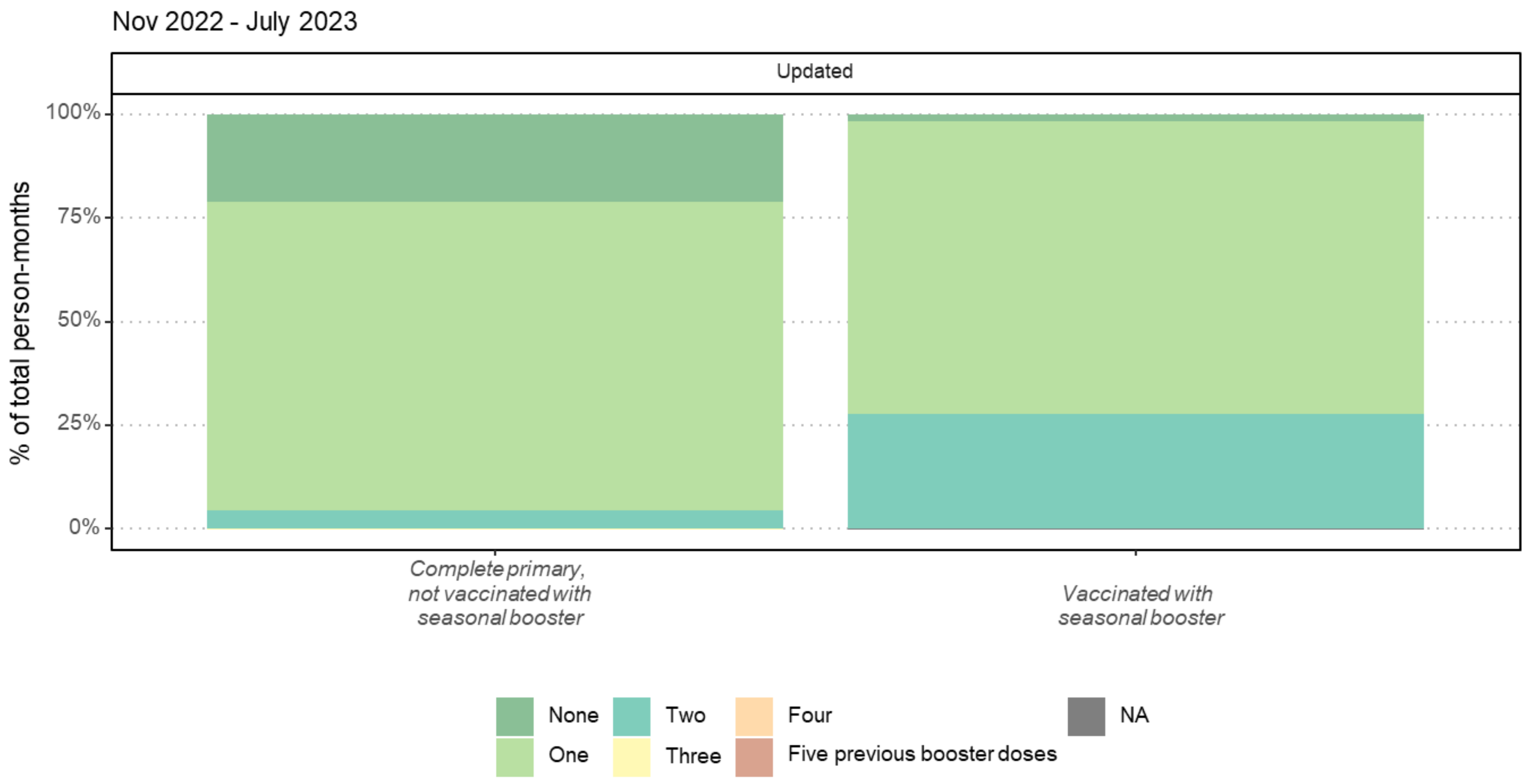

Figure S7b. Proportion of person-months (%) split by number of booster doses received previously and vaccination status, 65–79 years, November 2022-July 2023.

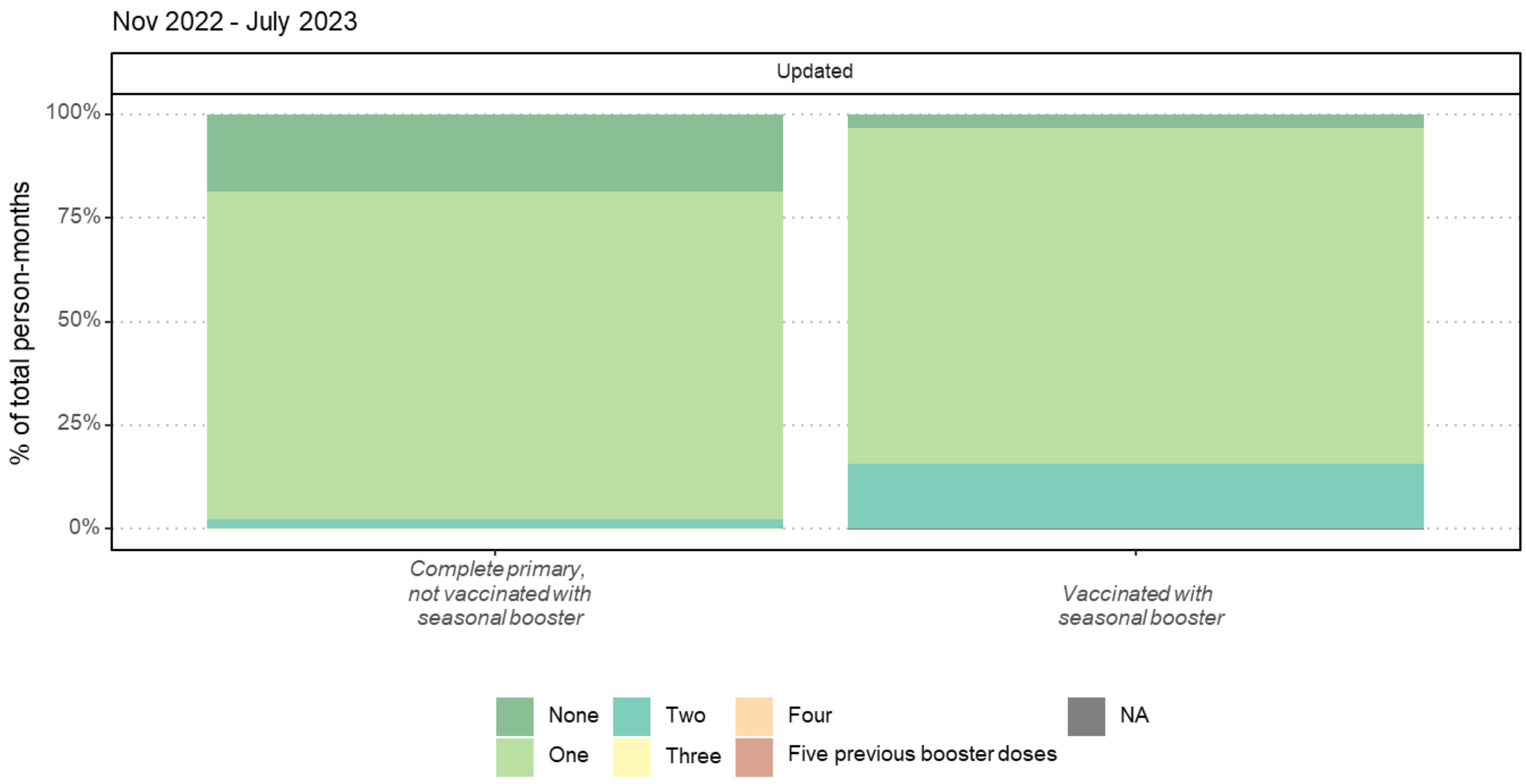

Figure S8a. Proportion of person-months (%) split by vaccine product received and vaccination status, 80 years, November 2022-July 2023.

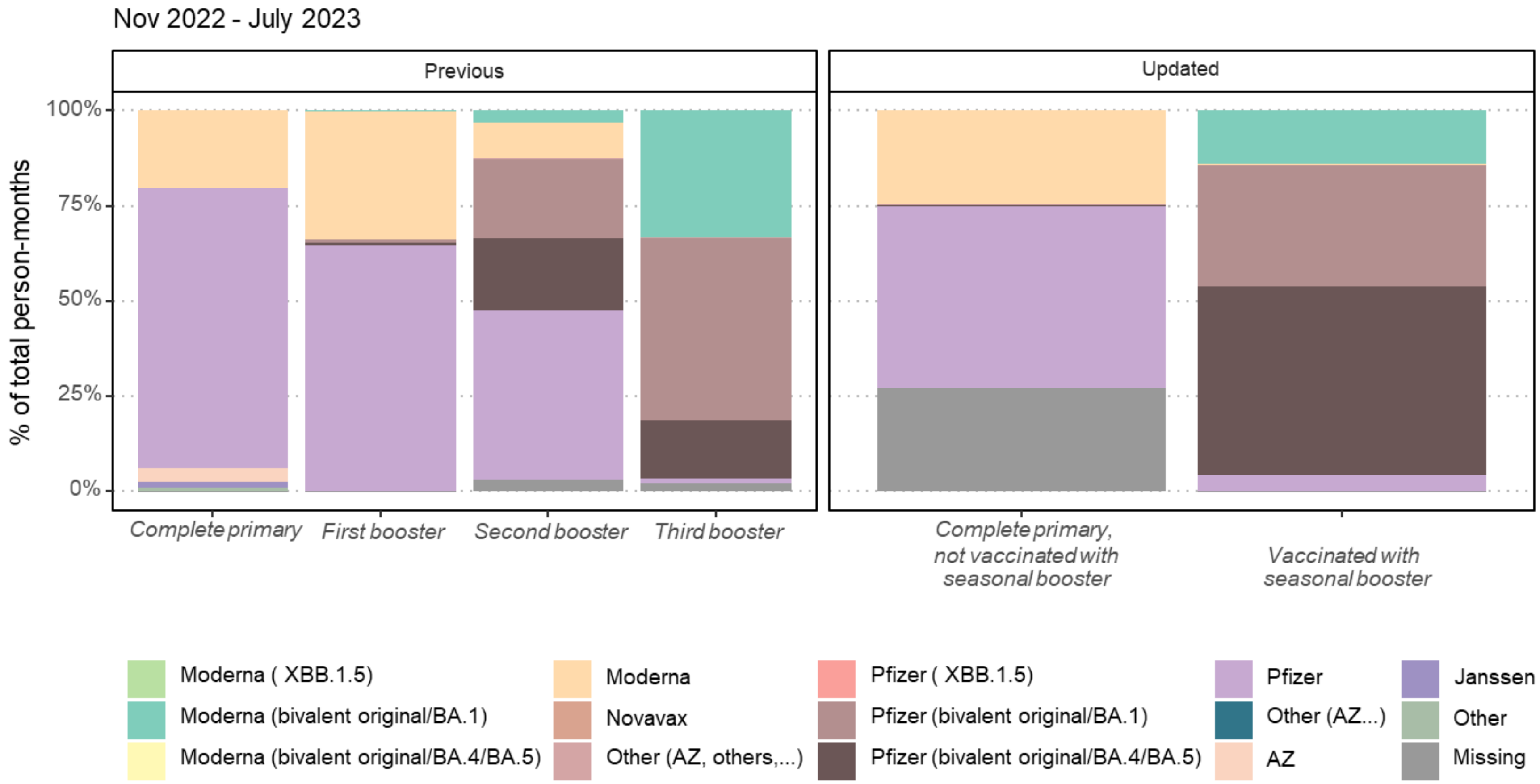

Figure S8b. Proportion of person-months (%) split by vaccine product received and vaccination status, 65–79 years, November 2022-July 2023.

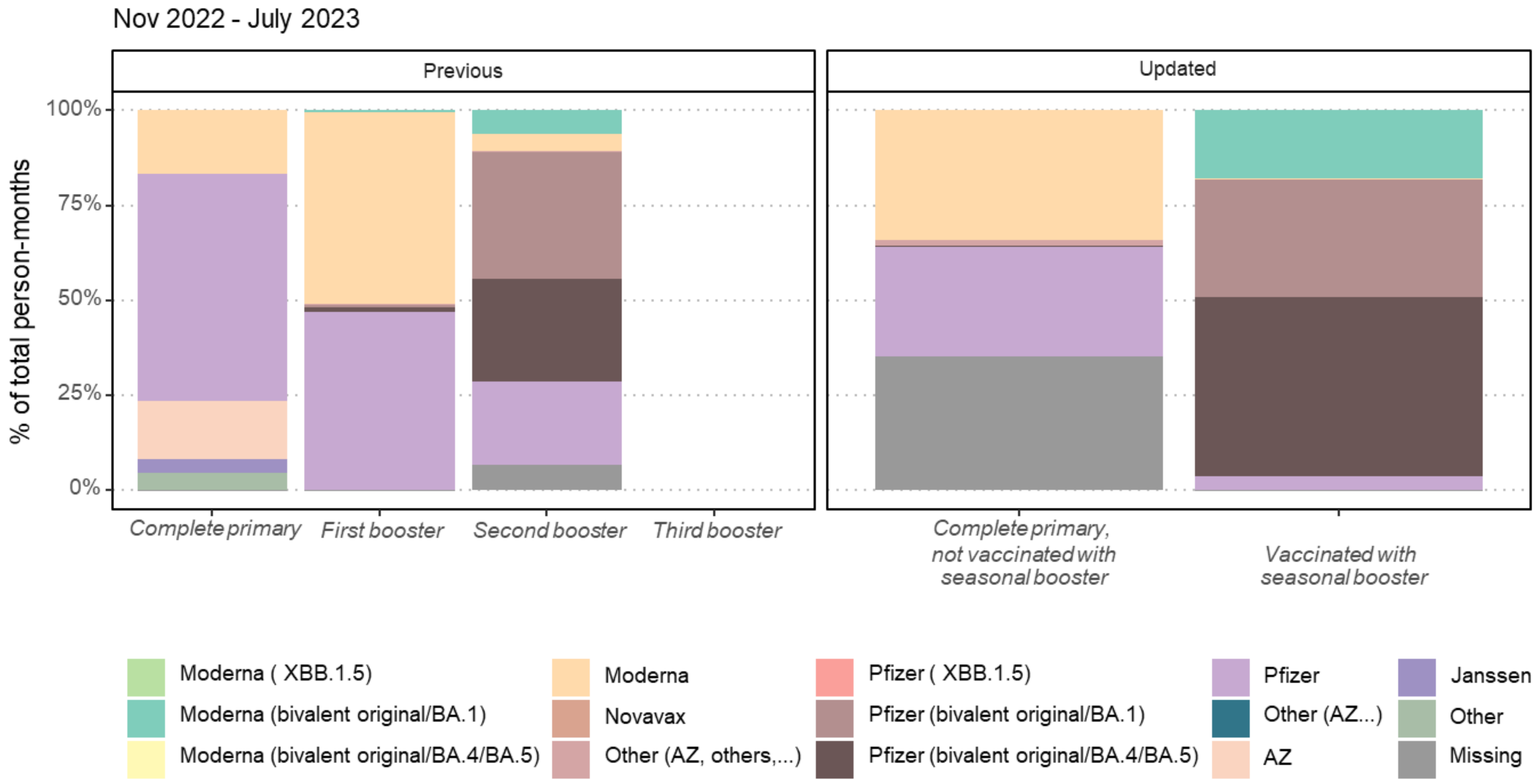

Annex 6. Vaccine effectiveness against COVID-19 hospitalisation by time since vaccination

Table S6. Relative vaccine effectiveness (95% confidence intervals) by number of weeks since vaccination in those aged ≥80 years against hospitalisation due to COVID-19 of the first, second and third booster dose, compared to complete primary vaccination without booster administered ≥24 weeks ago, by time since the booster, according to two approaches. First methodological approach: vaccine effectiveness (VE) of the first, second and third booster dose, compared to complete primary vaccination without booster administered ≥24 weeks ago. Second methodological approach: annual (bivalent) vaccine effectiveness among individuals eligible for annual vaccine. VE estimates for each 8-week overlapping study period, November 2022-July 2023.

Study period	First methodological approach									Second methodological approach		
	Complete primary vaccination + first booster dose			Complete primary vaccination + two booster doses			Complete primary vaccination + three booster doses			Seasonal vaccine booster		
	<12 weeks	12-<24 weeks	≥24 weeks	<12 weeks	12-<24 weeks	≥24 weeks	<12 weeks	12-<24 weeks	≥24 weeks	<12 weeks	12-<24 weeks	≥24 weeks
November 1 to December 26, 2022	51.6% (-429.8; 95.6)	13.3% (-111.5; 64.5)	16.6% (-7.7; 35.4)	65.7% (45.3; 78.4)	38.1% (1.4; 61.1)	-30.7% (-54.7; -10.3)	55.8% (41.4; 66.7)	-	-	62.8% (56; 68.5)	61.7% (48.3; 71.6)	-
December 1, 2022 to January 25, 2023	-38.6% (-103.7; 5.7)	14.4% (-502.1; 87.8)	10.8% (-14.1; 30.2)	57.6% (35.1; 72.3)	47.6% (12.3; 68.8)	-3.7% (-50.2; 28.4)	47.7% (25.7; 63.2)	53.0% (38.4; 64.2)	-	55.2% (48; 61.5)	50.3% (42.5; 57.1)	40% (-10; 67.3)
January 1 to Feb. 25,2023	-	-79.4% (-178.9; -15.4)	7.2% (-27.3; 32.4)	48.2% (24.1; 64.6)	47.9% (5.1; 71.4)	9.7% (-37.3; 40.6)	44.5% (13; 64.7)	16.2% (-39.2; 49.5)	-	44.7% (36.1; 52.1)	40.2% (30.2; 48.8)	37% (4.8; 58.3)
February 1 to March 28, 2023	-	-	-8.0% (-42.4; 18.2)	10.7% (-11.7; 28.6)	28.8% (-2; 50.2)	-2.5% (-55.4; 32.4)	40.1% (-45.1; 75.3)	3.5% (-76.9; 47.4)	48.2% (14; 68.8)	41.9% (10.5; 62.3)	26.7% (18.3; 34.3)	34.7% (20.6; 46.3)
March 1 to April 25, 2023	-	-81.7% (-206.1; -7.8)	10.9% (-27; 37.5)	19.1% (-8.1; 39.4)	32.9% (4.6; 52.8)	1.2% (-52.8; 36.2)	31.4% (-80.9; 74)	-	8.8% (-153.2; 67.1)	-2.1% (-105; 49.2)	25.7% (19.5; 31.4)	25.4% (16.8; 33.2)
April 1 to May 26, 2023	-	-165.8% (-341.8; -59.9)	11.3% (-30.8; 39.9)	-	36.4% (4.4; 57.7)	26.3% (-16.8; 53.4)	56.2% (-64; 88.3)	-0.8% (-42.2; 28.5)	1.5% (-67.3; 42.1)	29.5% (-21.9; 59.2)	23.9% (14.7; 32.1)	22.4% (10.9; 32.3)
May 1 to June 25, 2023	-	-	17.6% (-53.4; 55.7)	-	-16.4% (-57.5; 14)	14.4% (-38.7; 47.2)	60.2% (-237.3; 95.3)	-69.3% (-319.2; 31.6)	-8.1% (-76.6; 33.9)	-	28.9% (-18.6; 57.4)	19.8% (1; 35)
June 1 to July 26, 2023	-	-	14.8% (-38.4; 47.6)	-	-	16.0% (-26.2; 44)	-	-	15.8% (-41.8; 50)	-	-	13.7% (-3.5; 28.1)

Table S7. Vaccine effectiveness (95% confidence intervals) by number of weeks since vaccination in those aged 65–79 years against hospitalisation due to COVID-19 of the first, second and third booster dose, compared to complete primary vaccination without booster ≥24 weeks ago, by time since the booster, according to two approaches. First methodological approach: vaccine effectiveness (VE) of the first, second and third booster dose, compared to complete primary vaccination without booster administered ≥24 weeks ago. Second methodological approach: annual (bivalent) vaccine effectiveness among individuals eligible for annual vaccine. VE estimates for each 8-week overlapping study period, November 2022-July 2023.

Study period	First methodological approach									Second methodological approach		
	Complete primary vaccination + first booster dose			Complete primary vaccination + two booster doses			Complete primary vaccination + three booster doses			Seasonal vaccine booster		
	<12 weeks	12-<24 weeks	≥24 weeks	<12 weeks	12-<24 weeks	≥24 weeks	<12 weeks	12-<24 weeks	≥24 weeks	<12 weeks	12-<24 weeks	≥24 weeks
November 1 to December 26, 2022	19.0% (-21; 45.7)	-10.0% (-41.3; 14.4)	28.1% (22.4; 33.5)	72.7% (64.8; 78.8)	51.3% (24.9; 68.4)	-5.9% (-46; 23.2)	53.0% (17.4; 73.3)	-	-	66.2% (59.6; 71.7)	58% (22.7; 77.2)	-
December 1, 2022 to January 25, 2023	24.2% (-14.7; 49.9)	-3.3% (-97.3; 45.9)	28.4% (21.1; 34.9)	67.1% (52.6; 77.1)	55.7% (35.7; 69.4)	10.9% (-76; 54.9)	57.2% (35.2; 71.7)	48.5% (20.4; 66.7)	-	57.7% (47.4; 66)	46.9% (38.4; 54.2)	-
January 1 to Feb. 25,2023	-	8.8% (-32.4; 37.2)	33.4% (26.1; 40)	62.0% (55.8; 67.4)	54.0% (41.3; 63.9)	24.8% (2.2; 42.2)	58.8% (29.4; 75.9)	57.3% (42.9; 68.1)	-	46.4% (39.9; 52.2)	40.1% (33.7; 45.9)	14% (-43.5; 48.5)
February 1 to March 28, 2023	-	51.1% (23; 68.9)	20.2% (10.3; 29)	55.6% (44.8; 64.3)	48.1% (40.1; 55.1)	19.9% (7.7; 30.5)	57.5% (19.1; 77.6)	31.5% (-33.7; 64.9)	-	43.7% (31.8; 53.5)	42.8% (32.3; 51.6)	38.2% (18.3; 53.2)
March 1 to April 25, 2023	-	8.8% (-119.3; 62.1)	15.3% (4.4; 24.9)	55.8% (37.5; 68.8)	45.7% (36.4; 53.7)	32.8% (10; 49.8)	64.7% (41.3; 78.8)	24.8% (-59.8; 64.6)	51.3% (26.3; 67.8)	50.6% (26.6; 66.7)	37.2% (29.6; 44)	33% (21.7; 42.6)
April 1 to May 26, 2023	-	-	13.4% (0.4; 24.8)	52.6% (19.5; 72.1)	42.1% (23.1; 56.3)	39.1% (12.7; 57.6)	-	39.0% (-21.8; 69.4)	53.5% (30.4; 69)	40.4% (-11.2; 68.1)	31.6% (22.9; 39.3)	28.6% (20; 36.2)
May 1 to June 25, 2023	-	-	10.8% (-8.2; 26.6)	-	25.9% (0.2; 45)	19.2% (0.6; 34.4)	-	17.5% (-34.7; 49.5)	-0.4% (-167.7; 62.4)	-	19.6% (-16.4; 44.5)	29.1% (16.8; 39.6)
June 1 to July 26, 2023	-	-	26.8% (4; 44.2)	-	-	30.6% (3.4; 50.1)	-	-	6.3% (-71.1; 48.7)	-	7.4% (-69.8; 49.5)	22.8% (6.7; 36.2)

Annex 7. Vaccine effectiveness against COVID-19 mortality, overall

Table S8. Vaccine effectiveness (95% confidence intervals) in those aged ≥80 years against COVID-19 related death according to two approaches. First methodological approach: vaccine effectiveness (VE) of the first, second and third booster dose, compared to complete primary vaccination without booster administered ≥24 weeks ago. Second methodological approach: annual (bivalent) vaccine effectiveness among individuals eligible for annual vaccine. VE estimates for each 8-week overlapping study period, November 2022-July 2023.

Study period	First methodological approach			Second methodological approach
	Complete primary vaccination + first booster dose	Complete primary vaccination + two booster doses	Complete primary vaccination + three booster doses	Seasonal booster dose
November 1 to December 26, 2022	14.0% (-38.9; 46.7)	60.5% (9.6; 82.7)	56.8% (43.1; 67.2)	67.6% (61.7; 72.5)
December 1, 2022 to January 25, 2023	7.4% (-51.3; 43.3)	51.3% (-10.5; 78.6)	47.4% (26; 62.6)	60.0% (46.6; 70.1)
January 1 to Feb. 25,2023	11.0% (-39.8; 43.4)	51.0% (7.1; 74.1)	34.6% (8.9; 53)	51.0% (39.3; 60.4)
February 1 to March 28, 2023	-5.7% (-67.8; 33.4)	46.3% (-19.5; 75.9)	-5.5% (-51.5; 26.5)	44.0% (24.5; 58.5)
March 1 to April 25, 2023	-3.3% (-62.2; 34.2)	33.1% (-47; 69.5)	12.1% (-56.6; 50.7)	40.0% (24.8; 52.2)
April 1 to May 26, 2023	24.3% (-93.4; 70.4)	34.1% (-82; 76.2)	-4.2% (-77.9; 39)	29.1% (14.1; 41.4)
May 1 to June 25, 2023	-11.9% (-158.5; 51.6)	-10.6% (-159.5; 52.8)	11.1% (-39.7; 43.4)	24.0% (6; 38.6)
June 1 to July 26, 2023	-30.8% (-625.6; 76.4)	-8.8% (-574.2; 82.4)	-172.8% (-2170.8; 67.2)	29.9% (8.5; 46.3)

Table S9. Vaccine effectiveness (95% confidence intervals) in those aged 65 to 79 years against COVID-19 related death according to two approaches. First methodological approach: vaccine effectiveness (VE) of the first, second and third booster dose, compared to complete primary vaccination without booster administered ≥24 weeks ago. Second methodological approach: annual (bivalent) vaccine effectiveness among individuals eligible for annual vaccine. VE estimates or each 8-week overlapping study period, November 2022-July 2023.

Study period	First methodological approach			Second methodological approach
	Complete primary vaccination + first booster dose	Complete primary vaccination + two booster doses	Complete primary vaccination + three booster doses	Seasonal booster dose
November 1 to December 26, 2022	34.4% (8.7; 52.9)	77.3% (54.8; 88.6)	-	75.3% (69.6; 80)
December 1, 2022 to January 25, 2023	32.3% (14.9; 46.1)	74.9% (51.1; 87.1)	-	73.3% (62.5; 81)
January 1 to Feb. 25,2023	25.2% (-13.8; 50.8)	65.6% (52.5; 75.1)	-	67.8% (57.1; 75.7
February 1 to March 28, 2023	44.4% (14.6; 63.8)	68.7% (49.8; 80.4)	-	66.0% (53.8; 75)
March 1 to April 25, 2023	47.7% (19.8; 65.9)	62.9% (38.7; 77.6)	-	54.8% (39.6; 66.2)
April 1 to May 26, 2023	49.7% (0.2; 74.7)	51.5% (9; 74.2)	-	35.0% (13.3; 51.3)
May 1 to June 25, 2023	48.0% (-6; 74.5)	30.6% (-22.2; 60.6)	-	26.3% (-6.5; 49)
June 1 to July 26, 2023	38.1% (-16.7; 67.1)	27.3% (-36.9; 61.4)	-	25.9% (-13.1; 51.5)

Annex 8. Vaccine effectiveness against COVID-19 mortality, by time since vaccination

Table S10. Vaccine effectiveness (95% confidence intervals) by weeks since vaccination in those aged ≥80 years against COVID-19 related death of the first, second and third booster dose, compared to complete primary vaccination without booster administered ≥24 weeks ago, according to two approaches. First methodological approach: vaccine effectiveness (VE) of the first, second and third booster dose, compared to complete primary vaccination without booster administered ≥24 weeks ago. Second methodological approach: annual (bivalent) vaccine effectiveness among individuals eligible for annual vaccine. VE estimates for each 8-week overlapping study period, November 2022-July 2023.

Study period	First methodological approach									Second methodological approach		
	Complete primary vaccination + first booster dose			Complete primary vaccination + two booster doses			Complete primary vaccination + three booster doses			Seasonal vaccine booster		
	<12 weeks	12-<24 weeks	≥24 weeks	<12 weeks	12-<24 weeks	≥24 weeks	<12 weeks	12-<24 weeks	≥24 weeks	<12 weeks	12-<24 weeks	≥24 weeks
November 1 to December 26, 2022	-	2.8% (-609.4; 86.7)	-1.2% (-47; 30.3)	69.3% (35.1; 85.4)	18.3% (-29.1; 48.3)	-22.5% (-47.8; -1.6)	56.7% (42.7; 67.3)	-	-	72.1% (65.6; 77.3)	61.0% (28.5; 78.7)	-
December 1, 2022 to January 25, 2023	-	-90.3% (-219.1; -13.5)	-3.3% (-61.5; 33.9)	56.7% (24.1; 75.3)	36.0% (-44.9; 71.8)	-26.8% (-52; -5.7)	49.1% (31.4; 62.3)	30.0% (-10.7; 55.7)	-	67.2% (51.5; 77.8)	53.8% (45.4; 60.9)	-
January 1 to Feb. 25,2023	-	-	-2.6% (-55.3; 32.2)	43.7% (-23.1; 74.2)	39.0% (-19.7; 69)	-11.8% (-57.9; 20.9)	37.4% (9.3; 56.8)	37.0% (0.4; 60.2)	-	48.9% (22.6; 66.3)	53.9% (44.8; 61.6)	37.0% (-12.7; 64.8)
February 1 to March 28, 2023	-	-	-16.2% (-65.1; 18.2)	14.0% (-88.6; 60.8)	40.1% (-13.5; 68.4)	23.1% (-131.1; 74.4)	-20.4% (-120.8; 34.4)	-25.2% (-160.5; 39.8)	-	31.9% (-66.5; 72.1)	48.5% (25.2; 64.6)	34.6% (7.2; 54)
March 1 to April 25, 2023	-	-	-7.4% (-74.4; 33.9)	-	41.0% (-9.2; 68.1)	7.0% (-154; 66)	-100.4% (-350.2; 10.8)	-14.4% (-338.3; 70.2)	-99.0% (-364.9; 14.8)	-	46.7% (19.7; 64.6)	36.2% (20.4; 48.8)
April 1 to May 26, 2023	-	-	22.6% (-95.6; 69.4)	-	14.7% (-204; 76.1)	33.3% (-81.9; 75.5)	-	-	-61.3% (-333.5; 40)	-	30.4% (6.7; 48)	25.3% (7.8; 39.5)
May 1 to June 25, 2023	-	-	-12.5% (-159.5; 51.2)	-	-42.1% (-205.3; 33.9)	-13.7% (-167.5; 51.7)	-	-	14.0% (-42.6; 48.1)	-	19.5% (-26.1; 48.6)	24.8% (6; 39.9)
June 1 to July 26, 2023	-	-	-32.2% (-622.7; 75.8)	-	-	-11.8% (-579.1; 81.6)	-	-	-213.4% (-3242.4; 70.6)	-	-	28.9% (7; 45.7)

Table S11. Vaccine effectiveness (95% confidence intervals) by weeks since vaccination in those aged 65–79 years against COVID-19 related death of the first, second and third booster dose, compared to complete primary vaccination without booster ≥24 weeks ago, according to two approaches. First methodological approach: vaccine effectiveness (VE) of the first, second and third booster dose, compared to complete primary vaccination without booster administered ≥24 weeks ago. Second methodological approach: annual (bivalent) vaccine effectiveness among individuals eligible for annual vaccine. VE estimates for each 8-week overlapping study period, November 2022-July 2023.

Study period	First methodological approach						Second methodological approach		
	Complete primary vaccination + first booster dose			Complete primary vaccination + two booster doses			Seasonal vaccine booster		
	<12 weeks	12-<24 weeks	≥24 weeks	<12 weeks	12-<24 weeks	≥24 weeks	<12 weeks	12-<24 weeks	≥24 weeks
November 1 to December 26, 2022	-	-	27.5% (-24.2; 57.7)	77.7% (70.1; 83.3)	60.6% (24.5; 79.5)	-93.6% (-184.2; -31.8)	76.6% (68.1; 82.9)	69.0% (51.5; 80.2)	-
December 1, 2022 to January 25, 2023	-	-	18.3% (-20.6; 44.6)	76.0% (66.9; 82.6)	60.2% (45.2; 71)	-49.2% (-121.1; -0.6)	75.3% (62.9; 83.6)	72.1% (61.9; 79.6)	-
January 1 to Feb. 25,2023	-	-	-11.3% (-130.3; 46.2)	72.0% (47.2; 85.2)	61.0% (25.1; 79.7)	22.1% (-69.9; 64.3)	66.9% (41.1; 81.4)	69.0% (57.9; 77.3)	-
February 1 to March 28, 2023	-	-	-26.7% (-209.9; 48.2)	-	62.9% (-89; 92.7)	49.0% (14.6; 69.5)	-	65.9% (52.8; 75.4)	-
March 1 to April 25, 2023	-	-	42.5% (11.4; 62.7)	-	80.2% (60.4; 90.1)	50.2% (16.1; 70.5)	-	55.3% (33.5; 69.9)	41.4% (11.8; 61)
April 1 to May 26, 2023	-	-	47.1% (-0.9; 72.3)	-	57.0% (23.3; 75.9)	49.9% (1.1; 74.7)	-	39.9% (10.1; 59.8)	34.1% (8.3; 52.7)
May 1 to June 25, 2023	-	-	46.8% (-3; 72.5)	-	-	28.0% (-27.1; 59.2)	-	-	24.5% (-17.1; 51.4)
June 1 to July 26, 2023	-	-	37.4% (-18.5; 66.9)	-	-	29.1% (-34.1; 62.5)	-	-	23.3% (-16.6; 49.5)
